# Characterization of the RpoN regulon reveals the regulation of motility, T6SS2 and metabolism in *Vibrio parahaemolyticus*

**DOI:** 10.3389/fmicb.2022.1025960

**Published:** 2022-12-22

**Authors:** Dan Gu, Youkun Zhang, Kangru Wang, Mingzhu Li, Xinan Jiao

**Affiliations:** ^1^Jiangsu Key Laboratory of Zoonosis/Jiangsu Co-innovation Center for Prevention and Control of Important Animal Infectious Diseases and Zoonoses, Yangzhou University, Yangzhou, China; ^2^Key Laboratory of Prevention and Control of Biological Hazard Factors (Animal Origin) for Agrifood Safety and Quality, Ministry of Agriculture of China, Yangzhou University, Yangzhou, China; ^3^Joint International Research Laboratory of Agriculture and Agri-product Safety of the Ministry of Education, Yangzhou University, Yangzhou, China

**Keywords:** RpoN, motility, T6SS2, metabolism, *Vibrio parahaemolyticus*

## Abstract

*Vibrio parahaemolyticus* is a foodborne pathogen that can colonize the small intestine of the host and cause diarrhea. The alternative sigma factor RpoN plays a vital role in regulating motility, carbon utilization and affects host colonization in *V. parahaemolyticus* RIMD2210633. In this study, transcriptome and phenotypic analysis further expanded our understanding of the RpoN regulon in *V. parahaemolyticus*. A deletion mutant of *rpoN* (Δ*rpoN*) was subjected to RNA-seq for systemic identification of the RpoN-controlled genes. Compared with the wild-type (WT), 399 genes were differentially expressed in the Δ*rpoN* strain. Moreover, 264 genes were down-regulated in the Δ*rpoN* strain, including those associated with nitrogen utilization (*VP0118*), glutamine synthetase (*VP0121*), formate dehydrogenase (*VP1511* and *VP1513*-*VP1515*), quorum sensing (*opaR* and *luxZ*), polar flagellar systems, and type VI secretion system 2 (T6SS2). Quantitative real-time reverse transcription PCR (qRT-PCR) and electrophoretic mobility shift assay (EMSA) further confirmed that RpoN could directly bind to the promoters of these genes associated with polar flagellar systems (*flgB* and *fliE*), lateral flagellar systems (*flgB2* and *lafA*), T6SS2 (*hcp2* and *VPA1044*) and glutamine synthetase (*VP0121*), and then positively regulate the expression of these systems. A RpoN-binding motif was identified in *V. parahaemolyticus* using the MEME suite and verified by the EMSA. Besides, the deletion of *rpoN* caused a significant decrease in hemolytic activity, adhesion, and cytotoxicity. Our results provide new cues to better understand the regulatory networks of RpoN protein to motility, T6SS2, and metabolism in *V. parahaemolyticus*.

## Introduction

*Vibrio parahaemolyticus* is a halophilic Gram-negative bacterium that can cause seafood-borne gastroenteritis due to the consumption of raw or uncooked seafood in Japan, the United States, Brazil, and China (Nair et al., 2007; Raszl et al., 2016; Chen et al., 2017). Gastrointestinal pathogens can overcome the host immune defense system and environmental stresses, which is closely linked to their virulence. The main virulence of *V. parahaemolyticus* are thermostable direct hemolysin (TDH), TDH-related hemolysin (TRH), motility, biofilm, type III secretion system (T3SS1 and T3SS2) and type VI secretion system (T6SS1 and T6SS2; Love, 2017; Li et al., 2019), which are tightly regulated by the sigma factors and transcriptional regulators (Liu et al., 2021a; Zhang et al., 2021a). Besides, the environmental and intestinal factors can act as cues to induce the expression of genetic components involved in bacterial survival and virulence (Merrell and Camilli, 2002; Cai et al., 2021; Yin et al., 2021). Sigma factors are the most commonly identified transcriptional regulators that could sensor the environmental signals and interact with specific double-stranded DNA promoters of the genes to regulate the expression of these genes responsible for overcoming environmental stresses and conferring virulence (Davis et al., 2017; Wang et al., 2021; Wu et al., 2021).

RpoN is an alternative sigma factor that belongs to the sigma 54 families. This protein contains three domains: a DNA-enhancer-binding domain, an effector ATPase domain, and a receiver domain that could be phosphorylated to respond to environmental signals (Danson et al., 2019; Soules et al., 2020). RpoN protein acts as an alternative sigma factor in transcribing genes with diverse physiological roles in different bacteria. RpoN can recognize the −24 (GG) and −12(GC) elements in promoter regions and regulate the expression of genes responsible for metabolism and virulence, which is conserved in *E. coli*, *Vibrio cholerae*, and *Pseudomonas aeruginosa* (Zhao et al., 2010; Dong and Mekalanos, 2012; Shao et al., 2018; Liu et al., 2021b). In *V. cholerae*, RpoN can positively regulate the expression of 144 genes in 82 operons, including motility, T6SS, nitrogen utilization, formate dehydrogenase synthesis and phage shock protein synthesis (Dong and Mekalanos, 2012). Notably, 37 RpoN-controlled operons contain the conserved −24 and −12 elements in *V. cholerae* (Dong and Mekalanos, 2012). In *P. aeruginosa*, RpoN positively regulated 133 genes involved in translation, motility, protein folding, secondary metabolite biosynthetic process, T6SS, and QS system (Shao et al., 2018). Moreover, RpoN can directly bind to promoters in QS (*lasI*, *rhlI*, and *pqsR*) and T6SS (*hcpA* and *hcpB*), thereby affecting the functions of QS and T6SS in *P. aeruginosa* (Shao et al., 2018; Lloyd et al., 2019). In *V. parahaemolyticus*, the phenotypical analysis showed that RpoN plays an essential role in motility, utilization of carbon source (mucus) and affects host colonization (Whitaker et al., 2014). However, the regulon of RpoN has not been fully defined in *V. parahaemolyticus*.

Loss of *rpoN* enhances the colonization of *V. parahaemolyticus* in part due to the decrease of motility in the Δ*rpoN* strain (Whitaker et al., 2014). Furthermore, bacterial motility plays an essential role in colonization, adhesion, and biofilm formation (Brescia et al., 2020; Khan et al., 2020; Buchner et al., 2021; Mea et al., 2021). *V. parahaemolyticus* contains dual flagellar systems to adapt to different circumstances: the polar flagellar system was used when growing in liquids and the lateral flagellar system was used when growing on surfaces or in dense environments (McCarter, 2004). The expression of flagellar genes is highly regulated by QS, c-di-GMP, and transcriptional regulators in *V. parahaemolyticus* (Khan et al., 2020). Loss of RpoN leads to reduced motility in *V. parahaemolyticus* and other pathogens, including *V. cholerae*, *P. aeruginosa*, *Campylobacter jejuni*, *Yersinia pseudotuberculosis,* and *Shewanella baltica* (Syed et al., 2009; Whitaker et al., 2014; Lloyd et al., 2017; Mahmud et al., 2020; Sher et al., 2020; Fang et al., 2021).

Type VI secretion system is widespread in many Gram-negative bacteria, which could directly secrete the toxins to the other bacterial or eukaryotic cells (Mougous et al., 2006; Pukatzki et al., 2006; Bao et al., 2020). The *V. parahaemolyticus* contains two type VI secretion systems, T6SS1 and T6SS2 (Izutsu et al., 2008). T6SS1 is encoded by a gene cluster predominantly found in clinical isolates, which is active in warm marine-like conditions and deliver the effectors into the neighbour bacterial cell to mediate the antibacterial activity (Yu et al., 2012; Ceccarelli et al., 2013; Salomon et al., 2013). T6SS2 is found in all *V. parahaemolyticus* isolates, which could be active at cold temperatures and low salt conditions and contribute to the adhesion of *V. parahaemolyticus* to the HeLa cells (Wettstadt, 2020). The TfoY and the other two regulators, VP1391 and VP1407 could positively regulate T6SS1, whereas the H-NS negatively regulate T6SS1 (Ben-Yaakov and Salomon, 2019). T6SS2 is negatively regulated by H-NS and positively regulated by CalR (Salomon et al., 2014; Zhang et al., 2017a). In addition, the QS regulators AphA and OpaR also could directly regulate the expression of T6SS1 and T6SS2 (Zhang et al., 2017b).

This study focused on revealing the pathways regulated by RpoN in *V. parahaemolyticus*. RNA-seq was used to identify the regulon of RpoN followed by qRT-PCR validation. EMSA was then used to confirm the direct binding between RpoN and the promoter regions of polar flagellar gene clusters, lateral flagellar gene clusters, T6SS2, and metabolism-associated genes. Our results also confirmed the RpoN-controlled promoters, including *flgB*, *fliE*, *flgB2*, *lafK*, *hcp2*, *VPA1044*, and *VP0121*. Furthermore, *V. parahaemolyticus* phenotypic analysis indicated that RpoN could regulate the hemolytic activity, adhesion, and cytotoxicity. Our findings provided new insights into the detailed regulatory networks of the RpoN protein to metabolic and virulence-associated pathways in *V. parahaemolyticus*.

## Materials and methods

### Bacterial strains, plasmids, and growth conditions

The strains and plasmids used in the present study are listed in Supplementary Table S1. *V. parahaemolyticus* RIMD2210633 was used in the experiments. *E. coli* DH5α *λpir* and *E. coli* SM10 *λpir* were used for cloning and conjugation, respectively. All the *V. parahaemolyticus* and *E. coli* strains were cultured at 37°C in Luria-Bertani (LB) broth supplemented with 1% NaCl. The following antibiotics were added when required: carbenicillin (Carb, 100 μg/ml) and chloramphenicol (Cm, 40 μg/ml). In addition, Isopropyl β-D-1-thiogalactopyranoside (IPTG, 1 mmol/ml) was used to induce the expression of RpoN in *rpoN*^+^ and BL21/pET32a::*rpoN* strain.

### Construction of the *rpoN* in-frame deletion mutant and complemented strains

The *rpoN* (*VP2670*) in-frame deletion mutant strain was constructed according to a previous method using the suicide vector pDM4 (Zhou et al., 2010), the primers used were listed in Supplementary Table S2. In brief, the upstream and downstream fragments of *rpoN* were amplified from the genome of *V. parahaemolyticus* with primers *rpoN* up-F/R and *rpoN* down-F/R and cloned into pDM4 with Sac I/Sal I sites by a ClonExpress Multis One Step Cloning Kit (Vazyme, Nanjing, China). Then, the positive recombinant plasmid pDM4::Δ*rpoN* was transformed into the WT strain by conjugation and cultured on an LB agar plate containing Carb and Cm. Following this, the second cross-over recombination was detected in the LB agar plate with 15% sucrose. Finally, the *rpoN* deletion mutant strain was verified by PCR with the primers *rpoN* out-F/R and *rpoN* in-F/R and sequencing analysis.

The ORF of *rpoN* was amplified with primers *rpoN* com-F/R and cloned into the pMMB207 plasmid with Xba I/Hind III sites by a ClonExpress Multis One Step Cloning Kit (Vazyme, Nanjing, China). Then, the positive recombinant plasmid pMMB207::*rpoN* was transformed into the Δ*rpoN* strain and selected on an LB agar plate containing Carb and Cm. The complemented strain was confirmed by PCR with primers pMMB207-F/R and named *rpoN*^+^.

### RNA-seq analysis

The *V. parahaemolyticus* WT and ∆*rpoN* strains were cultured on LB agar plates for 15 h. One clone was picked and inoculated into LB broth growing at 37°C for 12 h. The cultured bacteria were diluted to 1:100 in new LB broth for 5–6 h at the late logarithmic growth phase (Supplementary Figure S4). Total RNA was extracted using the RNeasy Plus Mini Kit (Qiagen, Hilden, Germany). First strand cDNA was synthesized from the rRNA-depleted RNA samples and purified using an RNA Clean. After second strand cDNA synthesis, we performed end repair, 3′ end adenylation and adapter ligation, and the library was amplified by PCR. The three parallel RNA samples were sequenced using Illumina HiSeq (GENEWIZ, Suzhou, China). Statistical analysis was performed as described previously (Tang et al., 2021).

### qRT-PCR

The qRT-PCR was performed as previously described (Gu et al., 2020). The WT, Δ*rpoN*, Δ*rpoN*Δ*opaR*, Δ*rpoN*Δ*qrr2*, and Δ*rpoNopaR*^+^ strains were cultured at 37°C in LB medium overnight and diluted 1:100 in new LB medium for 5–6 h or in the BHI agar for 48 h. Total RNA was extracted using the RNeasy Plus Mini Kit (Qiagen). Genomic DNA was removed using RNase-free DNase I. Equal amounts of RNA (1 μg) were used to generate the first-strand cDNA using the PrimeScript RT Reagent Kit with a gDNA eraser (Takara, Tokyo, Japan). The specific primers used for qRT-PCR are listed in Supplementary Table S2. The reactions were performed on the ABI PRISM 7500 Real-Time PCR System (Applied Biosystems, Foster City, CA, United States) with a FastStart Universal SYBR Green Master (Roche, Mannheim, Germany). The transcript levels of each sample were normalized to those of *gyrB* using the 2^−ΔΔCt^ method. Three independent experiments were performed, and each experiment was run in triplicate.

### Motility assay

Overnight cultures of WT, Δ*rpoN*, *rpoN*^+^, Δ*rpoN*/pMMB207, Δ*rpoN*Δ*opaR*, Δ*rpoN*Δ*qrr2*, and Δ*rpoNopaR*^+^ strains were adjusted to an optical density at 600 nm (OD_600_) of 1.0, following which 5 μl volumes of the diluted cultures were spotted on different plates. LB medium with 0.3% agar was used for swimming motility assays, and BHI medium with 1.5% agar was used for swarming motility assays. The LB plates were cultured at the 37°C for 12 h, and the BHI plates were cultured at 30°C for 24 h. IPTG was used to induce the expression of RpoN protein in *rpoN*^+^ and Δ*rpoN*/pMMB207 strains. Each experiment was performed three times.

### Overexpression and purification of the RpoN protein

The ORF region of *rpoN* was amplified with primers RpoN-F/R and cloned into the pET32a plasmid with sites of XhoI and BamHI. The positive recombinant plasmid pET32a::*rpoN* was transformed into *E. coli* BL21 (DE3), and verified by PCR with primers pET32a-F/R. The specific primers are listed in Supplementary Table S2. BL21/pET32a::*rpoN* was cultured in LB broth, and IPTG was added to induce the expression of RpoN until the OD_600_ value was between 0.4 and 0.6. Then, the bacteria were cultured at 120 rpm and 16°C for 16 h. The cell cultures were collected, washed, lysed, and subjected to the purification of the RpoN-His protein using the His Bind Purification Kit (Novagen, Darmstadt, Germany).

### EMSA

EMSA was performed as described previously (Gu et al., 2016). The primers used for EMSA are listed in Supplementary Table S2. The DNA probes were amplified by the primers with the FAM fluorescent label and purified using the Agarose Gel DNA Extraction Kit (Tiangen, Beijing, China). Each EMSA reaction mixture (20 μl) consisted of 10 ng of DNA probes, 4 μl of EMSA buffer (10 mM NaCl, 0.1 mM DTT, 0.1 mM EDTA, 10 mM Tris, pH 7.4), 1 μl of poly-dIdC, different concentrations of the RpoN-His protein, and ddH_2_O. The mixture was incubated at 25°C for 0.5 h and separated on a 6% native PAGE gel. Finally, the gel was scanned using Typhoon FLA 9500 (GE Healthcare, Uppsala, Sweden).

### Adhesion assay

The adhesion assay was performed as described previously (Qiu et al., 2020). The cultures of WT, Δ*rpoN*, and *rpoN*^+^ strains were pelleted by centrifugation, washed, and resuspended in dulbecco phosphate-buffered saline (DPBS). HeLa cell monolayers were infected at a multiplicity of infection (MOI) of 1:100. After incubation at 37°C under 5% CO_2_ for 2 h, the HeLa cells were washed twice with DPBS and lysed with 0.01% Triton-X 100. The lysates and bacteria were serially diluted and counted on LB agar plates. The adherence rate was calculated as the number of bacterial cells adhered/the number of bacterial cells input.

### Hemolytic activity assay

Hemolytic activity was assessed as described previously (Rattanama et al., 2012; Kongrueng et al., 2018). Overnight cultures of WT, Δ*rpoN*, *rpoN*^+^, Δ*rpoN*/pMMB207, Δ*rpoN*Δ*opaR*, Δ*rpoN*Δ*qrr2*, and Δ*rpoNopaR*^+^ strains were centrifuged, washed thrice with PBS, and resuspended in DPBS at a final concentration of 0.5 × 10^9^ CFU/ml. Following this, 5 μl of the bacterial suspension was added to the wells in the blood agar plates and cultured at 37°C for 12–24 h. The diameter of clear zone around the well was indicated as the hemolytic activity. The experiments were repeated at least three times.

### Cytotoxicity assay

The infection of HeLa cells and the release of lactate dehydrogenase (LDH) were assessed as described previously (Erwin et al., 2012). Overnight cultures of WT, Δ*rpoN*, *rpoN*^+^, Δ*rpoN*/pMMB207, Δ*rpoN*Δ*opaR*, Δ*rpoN*Δ*qrr2*, and Δ*rpoNopaR*^+^ strains were diluted 1:100 in fresh LB medium and shaken at 37°C for 4 h. Following this, the cultures were centrifuged, and pellets were resuspended in dulbecco’s modified eagle medium (DMEM) containing 10% fatal bovine serum (FBS). The bacteria were diluted to a concentration of 10^8^ CFU, and the bacterial suspensions were inoculated into each well of a 12-well plate containing 10^6^ HeLa cells/well to achieve an MOI of 100 CFU/cell. After infection for 3 h, the LDH activity of the supernatants was measured using the LDH Cytotoxicity Assay Kit (Beyotime, Haimen, China), according to the manufacturer’s protocol.

## Results

### Identification the regulon of RpoN in *V. parahaemolyticus* RIMD2210633

**Figure 1:**
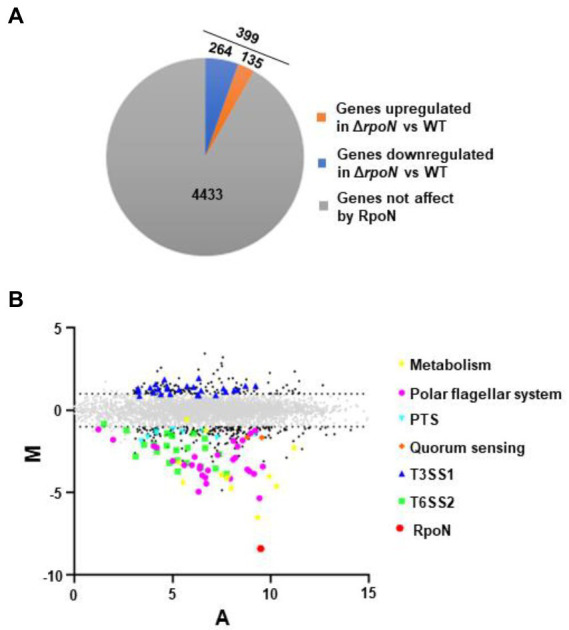
The regulon of RpoN in *V. parahaemolyticus.* **(A)** Pie charts show the genes whose expression significantly differed between Δ*rpoN* and WT strains in LB liquid medium. **(B)** MA plots showing changes that were observed in gene expression at Δ*rpoN* compared to WT. The genes associated with metabolism and virulence are highlighted. The log2 values for the ratios of abundances for each transcript are shown between Δ*rpoN* and WT (M, *y* axis) and plotted against the average log2 for abundance for that transcript under both conditions (A, *x* axis). Solid black dots, *P*_adj _< 0.05.

We assessed the regulon of RpoN in *V. parahaemolyticus* by the RNA-seq analysis. Comparison of the transcriptomes for the WT and Δ*rpoN* strains grown in LB medium revealed that the expression of 399 genes significantly differed between the Δ*rpoN* and WT strains (Log_2_Fold Change ≥1 or ≤−1, *p*-value < 0.05). As shown in Figure 1A, 135 and 264 genes were up-regulated and down-regulated in the Δ*rpoN* strain, respectively. As expected, *rpoN* was not detected in Δ*rpoN* and highly expressed in WT, suggesting that the RNA-seq data is reliable (Figure 1B). Figure 1B described the expression patterns of genes that are potentially associated with metabolic and virulence, including metabolism, phosphotransferase system (PTS), polar flagellar system, quorum sensing, T3SS1 and T6SS2.

Compared with the WT strain, the QS master regulator protein OpaR and the sensor protein LuxN were significantly down-regulated in the Δ*rpoN* strain. Furthermore, many carbon source-associated genes were significantly regulated by RpoN, such as the nitrogen regulatory protein (*VP0118*), glutamine synthetase (*VP0121*), formate dehydrogenase (*VP1511* and *VP1513*-*VP1515*), and PTS system (Table 1). In addition, virulence-associated genes were also regulated by RpoN. Compared with the WT, the genes related to T6SS2, flagellar systems, and biofilm formation were down-regulated, while those related to the type III secretion system 1 (T3SS1) and siderophore-dependent iron uptake systems were up-regulated in the Δ*rpoN* strain. The above results indicate that RpoN is an important sigma factor regulating metabolic and virulence-associated pathways in *V. parahaemolyticus*.

**Table 1:** The genes negatively regulated in the Δ*rpoN* compared to WT.

Gene name	Annotation	Log_2_foldchange (Δ*rpoN*/WT)	*p*-value	*V. cholerae* ChIP-seq (Dong and Mekalanos, 2012)*
**Carbon utilization**				
VP0118	nitrogen regulation protein	−1.21	1.83E-08	Y
VP0121	glutamine synthetase	−2.27	6.15E-86	Y
VP1510	(Fe-S)-binding protein	−4.02	1.29E-22	Y
VP1511	formate dehydrogenase-specific chaperone	−4.75	1.92E-71	
VP1512	hypothetical protein	−3.17	7.58E-10	
VP1513	formate dehydrogenase large subunit	−4.60	3.99E-151	
VP1514	formate dehydrogenase iron–sulfur subunit	−3.93	2.50E-22	
VP1515	formate dehydrogenase cytochrome b556 subunit	−4.05	9.61E-74	
**PTS**				
VP0366	phosphoenolpyruvate-protein phosphotransferase	−1.42	2.32E-21	
VP0710	PTS system trehalose(maltose)-specific transporter subunits IIBC	−1.68	0.002609259	
VP0711	trehalose-6-phosphate hydrolase	−1.26	0.025630694	
VP0810	PTS system mannose-specific factor IIC	−1.38	0.001487326	
VP2636	PTS system cellobiose-specific transporter subunit Iic transporter subunit	−1.31	0.001933048	
VP2637	PTS system cellobiose-specific transporter subunit IIB	−1.88	0.000168365	
VPA0811	PTS system fructose-specific transporter subunit IIBC	−1.31	1.78E-05	
VPA0812	1-phosphofructokinase	−1.61	0.000783696	
VPA0813	bifunctional PTS system fructose-specific transporter subunit IIA/HPr protein	−1.19	0.000866908	
**Flagellar**				
VP0775	flagellar basal-body rod protein FlgB	−2.19	5.27E-47	Y
VP0776	flagellar basal body rod protein FlgC	−2.71	5.43E-36	
VP0777	flagellar basal body rod modification protein	−2.88	9.16E-69	
VP0778	flagellar hook protein FlgE	−3.41	8.05E-105	Y
VP0780	flagellar basal body rod protein FlgF	−3.96	2.44E-35	
VP0781	flagellar basal body rod protein FlgG	−4.47	2.47E-44	
VP0782	flagellar basal body L-ring protein	−3.55	1.26E-34	
VP0783	flagellar basal body P-ring biosynthesis protein FlgA	−3.40	3.51E-34	Y
VP0784	flagellar rod assembly protein/muramidase FlgJ	−4.09	1.34E-44	
VP0785	flagellar hook-associated protein FlgK	−4.95	9.14E-38	Y
VP0786	flagellar hook-associated protein FlgL	−2.83	6.35E-24	
VP0788	flagellin	−3.86	1.11E-125	Y
VP0790	flagellin	−2.26	2.03E-07	
VP2229	chemotaxis protein CheA	−1.11	1.54E-15	
VP2232	flagellar biosynthesis sigma factor	−1.83	6.02E-40	
VP2233	flagellar biosynthesis protein FlhG	−3.71	3.51E-115	
VP2234	flagellar biosynthesis regulator FlhF	−5.35	6.38E-154	Y
VP2235	flagellar biosynthesis protein FlhA	−3.60	3.78E-78	Y
VP2244	polar flagellar hook-length control protein FliK	−1.86	4.68E-24	Y
VP2248	flagellar motor switch protein G	−1.40	4.17E-14	
VP2251	FlaM	−1.23	1.20E-18	
VP2254	flagellar protein FliS	−3.08	2.88E-16	
VP2256	flagellar capping protein	−3.66	5.73E-44	Y
VP2257	flagellar protein FlaG	−3.33	4.50E-28	
VP2258	flagellin	−3.00	2.95E-69	
VP2259	flagellin	−3.33	7.63E-23	
VP2261	flagellin	−2.17	8.90E-07	
**Quorum sensing**				
VP1968	sensor protein LuxN	−1.66	1.19E-37	
VP2516	OpaR protein	−1.64	1.41E-31	
**T6SS2**				
VPA1024	hypothetical protein	−1.40	9.00E-05	
VPA1025	hypothetical protein	−1.22	0.00406734	Y
VPA1026	hypothetical protein	−2.29	3.12E-22	Y
VPA1027	hypothetical protein	−3.85	2.82E-75	
VPA1028	ClpA/B-type chaperone	−1.40	6.23E-08	
VPA1029	hypothetical protein	−2.10	2.19E-05	
VPA1030	hypothetical protein	−1.56	1.11E-05	
VPA1032	hypothetical protein	−2.81	1.45E-07	
VPA1033	hypothetical protein	−2.08	2.22E-06	
VPA1034	hypothetical protein	−3.48	2.32E-29	
VPA1035	hypothetical protein	−2.52	2.76E-08	
VPA1036	hypothetical protein	−2.43	6.12E-13	
VPA1037	phosphoprotein phosphatase	−2.16	1.39E-07	
VPA1038	hypothetical protein	−3.21	1.84E-15	
VPA1039	hypothetical protein	−2.66	4.48E-40	
VPA1040	hypothetical protein	−3.03	3.40E-17	
VPA1041	hypothetical protein	−3.21	7.54E-23	
VPA1042	hypothetical protein	−3.71	4.16E-21	
VPA1043	hypothetical protein	−3.53	7.48E-51	
VPA1044	hypothetical protein	−3.43	2.03E-35	
VPA1045	hypothetical protein	−1.72	9.84E-15	
VPA1046	hypothetical protein	−2.27	1.48E-14	
**Other genes**				
VP0768	hypothetical protein	−1.96	1.62E-14	Y
VP1173	phage shock protein A	−1.01	4.36E-10	Y
VP1393	BfdA protein	−1.84	3.17E-05	Y
VP1501	hypothetical protein	−1.45	7.63E-14	Y
VP1508	hypothetical protein	−1.84	1.03E-08	Y
VP2162	hypothetical protein	−1.56	0.000476209	Y
VPA0188	hypothetical protein	−2.81	1.81E-08	Y

* Y means that this gene can be peaked in the data of RpoN ChIP-seq in *V. cholerae*.

**Figure 2:**
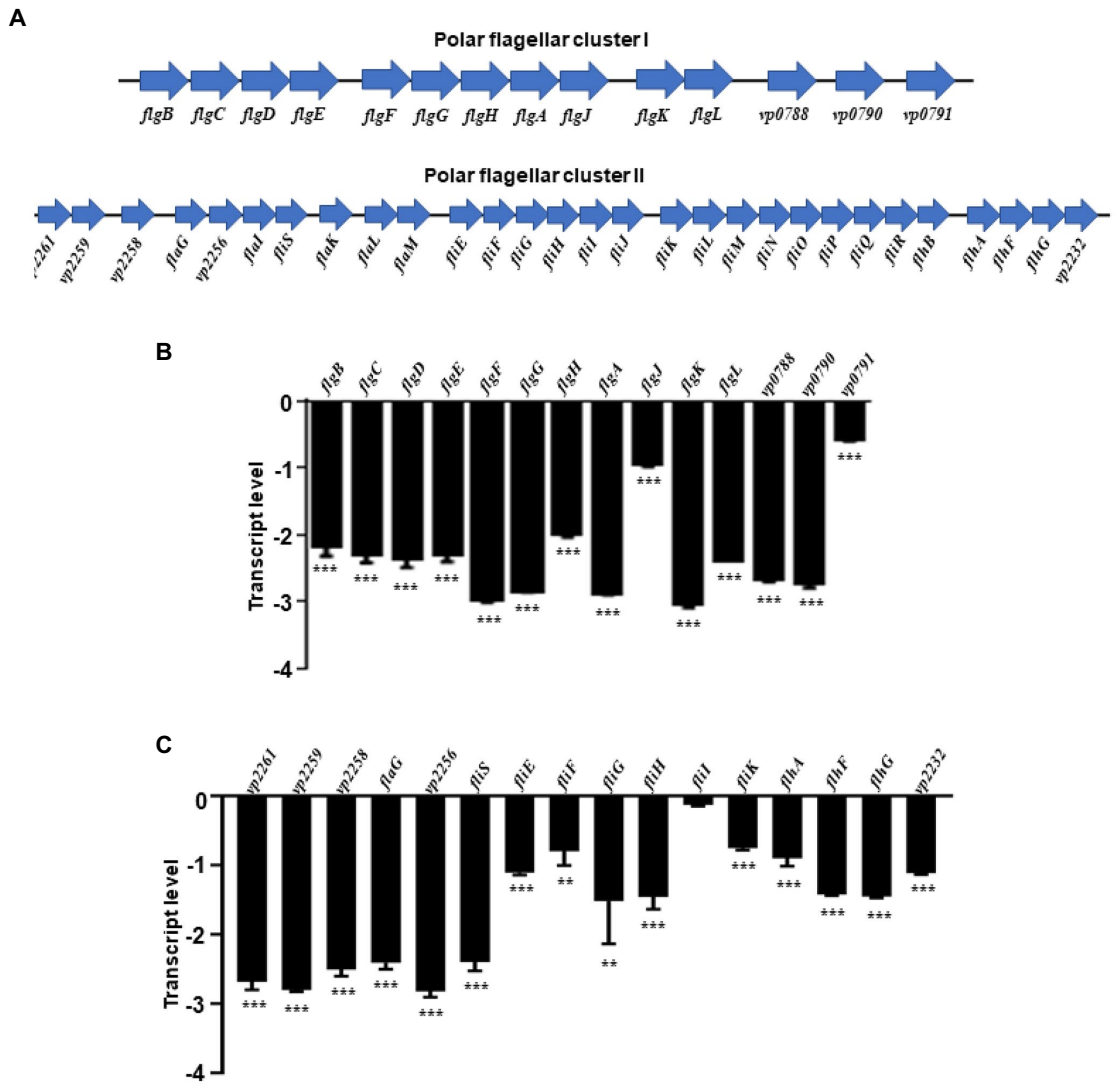
The RpoN positively regulates the expression of flagellar clusters. **(A)** Genetic map of polar flagellar cluster I and cluster II genes in *V. parahaemolyticus*. **(B,C)** qRT-PCR analysis of the transcription levels of polar flagellar cluster I genes **(B)** and polar flagellar cluster II genes **(C)**. The data are presented as the mean ± SD (*n* = 3). **p < 0.01, ***p < 0.001, Student’s *t* test analyzes Δ*rpoN* compared to WT.

**RpoN could regulate the expression of polar flagellar genes to mediate swimming motility in** ***V. parahaemolyticus***. RNA-seq results revealed that the polar flagellar genes were down-regulated in the Δ*rpoN* strain compared with the WT. Assessment of the polar flagellar gene clusters in *V. parahaemolyticus* revealed that 43 genes were divided into two clusters: polar flagellar gene cluster I (14 genes) and polar flagellar gene cluster II (29 genes; Figure 2A). The transcript levels of the two polar flagellar gene clusters identified by RNA-seq are shown in Table 1. All of the genes located in the polar flagellar cluster I were significantly down-regulated in the Δ*rpoN* strain. Some of the genes located in polar flagellar cluster II were also significantly down-regulated in the Δ*rpoN* strain, suggesting that the RpoN protein could positively regulate the expression of polar flagellar genes.

All of the polar flagellar cluster I genes and some of the cluster II genes were subjected to qRT-PCR analysis to verify the results of RNA-seq. All polar flagellar cluster I genes in the Δ*rpoN* strain were significantly down-regulated compared with the WT strain (Figure 2B). In particular, *VP2261*, *VP2259*, *VP2258*, *flaG*, *VP2256*, *fliS*, *fliEFGHI, fliK*, *flhAFG*, and *VP2232* located in polar flagellar cluster II were significantly down-regulated by RpoN (Figure 2C). Furthermore, motility analysis revealed that the swimming ability was lost in the Δ*rpoN* strain, while the swimming ability in *rpoN* complementary (*rpoN*^+^) strain was restored to the similar level observed in the WT (Supplementary Figure S1A), which is consistent with the previous study (Whitaker et al., 2014). Collectively, these results indicate that RpoN is a sigma factor that could regulate the expression of polar flagellar genes to mediate swimming motility in *V. parahaemolyticus*.

### RpoN directly binds to the promoters of the polar flagellar genes *flgB* and *fliE*

**Figure 3:**
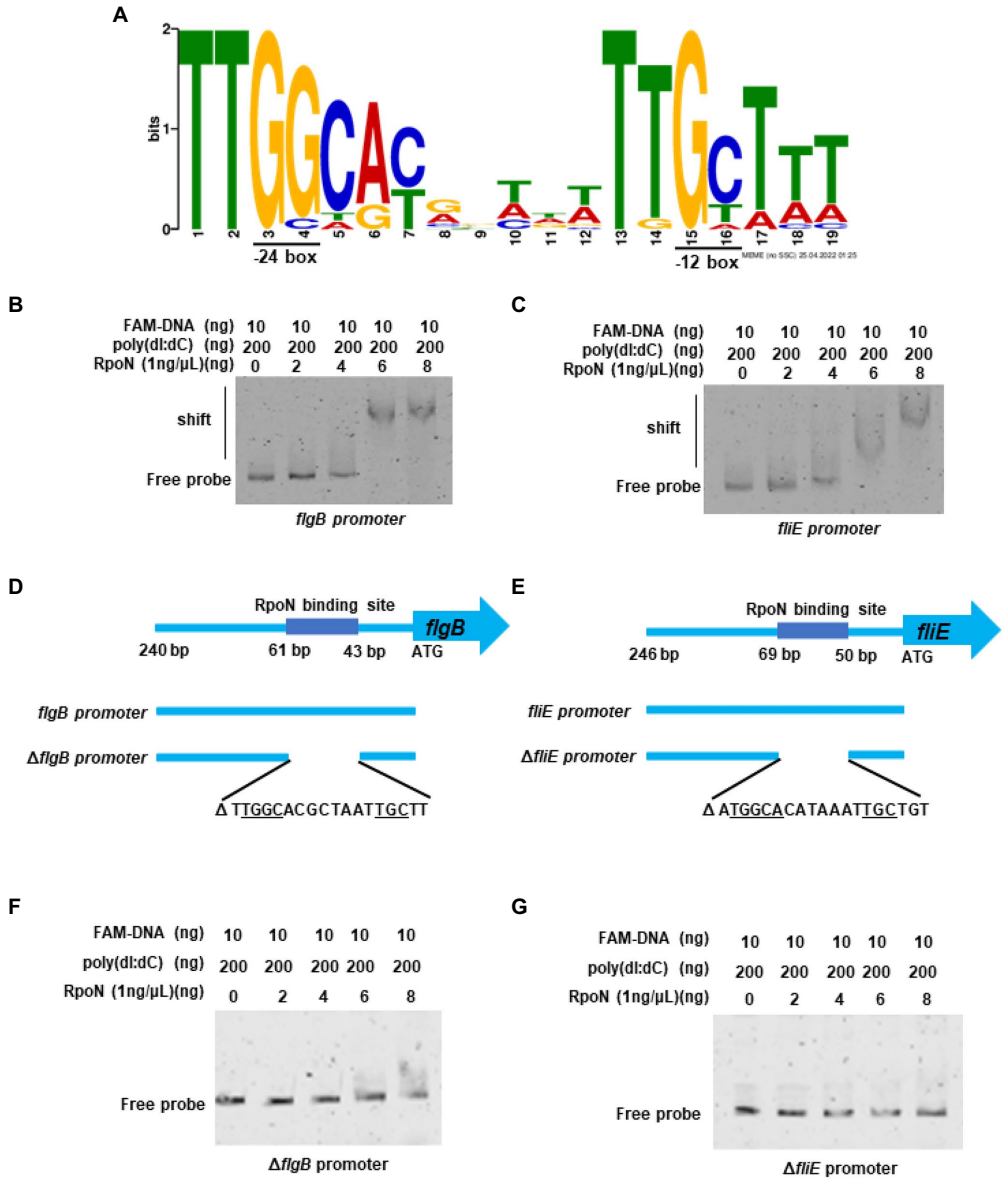
The RpoN directly binds to the promoters of *flgB* and *fliE*. **(A)** The binding motif of RpoN was generated by MEME-Suit tool based on the 21 promoter regions of the gene differentially expressed in our RNA-seq and overlapped with the ChIP-seq of *V. cholerae*. The height of each base indicates the frequency of occurrence at each location. **(B,C)** EMSA revealed that RpoN directly binds to the promoter regions of *flgB* **(b)** and *fliE* **(C)**. **(D)** The promoter region of the *flgB*. The RpoN binding sites and the probes of *flgB* promoter (240 bp) and the ∆*flgB* promoter with deletion of RpoN binding site ranging from bp 43–61 relative to start site ATG. **(E)** The promoter region of the *fliE*. The RpoN binding sites and the probes of *fliE* promoter (246 bp) and the ∆*fliE* promoter with deletion of RpoN binding site ranging from bp 50–69 relative to start site ATG. **(F,G)** EMSA was performed to assess the binding of RpoN to specific RpoN binding site-deleted promoters ∆*flgB* **(F)** and ∆*fliE* **(G)**.

In *V. cholerae*, 68 RpoN-binding peaks were identified by ChIP-seq (Dong and Mekalanos, 2012). Therefore, we used the 68 RpoN-binidng peaks blast against the genome of *V. parahaemolyticus* and 21 genes were found significantly down-regulated in Δ*rpoN* by RNA-seq (Table 1). Then, the promoter regions of these 21 genes were used to generate the RpoN-binding motif by MEME-Suit tool.^1^ As shown in Figure 3A, the −24 box (GG) and −12 box (GC) were identified as two conserved motifs, and 15 of 21 genes contained the conserved RpoN binding sites (Table 2). The *fliE* (*VP2250*) and *flaL* (*VP2252*) genes were no difference expressed between Δ*rpoN* and WT in RNA-seq, whereas peaked in ChIP-seq of *V. cholerae* and contained the conserved RpoN-binding sites in *V. parahaemolyticus* (Table 2). The promoters of *flgB* (*VP0775*), *flgF* (*VP0780*), *flgK* (*VP0785*), *flhA* (*VP2235*), *flaL* (*VP2252*), and *fliE* (*VP2250*) involved in the polar flagellar gene clusters were selected to confirm by EMSA. The results showed that RpoN could directly bind to the promoters of *flgB* (Figure 3B) and *fliE* (Figure 3C) in a concentration-dependent manner; however, it could not directly bind to the other promoters (Supplementary Figures S2A–D). RpoN did not bind to the promoter of *gyrB* at the highest concentration (Supplementary Figure S2E), which was used as a negative control.

**Table 2:** The conserved RpoN binding sites.

Gene name	Production	Binding motif
**Overlapped with ChIP-seq of** ***V. cholerae***		
VP0768	hypothetical protein	TTGGAACAGTCTTTGCTTT
VP0775	flagellar basal body rod protein FlgB	TTGGCACGCTAATTGCTTT
VP0780	flagellar basal body rod protein FlgF	TTGGCATAACTATTGCTTT
VP0785	flagellar hook-associated protein FlgK	TTGGCACATCTTTTGCTTT
VP0788	flagellin	TTGGCACACAAATTGTATT
VP2235	flagellar biosynthesis protein FlhA	TTGGTACATAGATTGCTTA
VP2244	polar flagellar hook-length control protein FliK	TTGGCGTGATTTTTGCAAA
VP0121	glutamine synthetase	TTGGCACGGTTTTGGCTTT
VP1173	phage shock protein A	TTGGCATGGTACTTGTTAT
VP1501	hypothetical protein	TTGGCATCTTGTTTGCTAT
VP1508	hypothetical protein	TTGGCGCGGTTATTGCTTT
VP1510	(Fe-S)-binding protein	TTGGCATGACATTTGCTAT
VP1393	bfdA protein	TTGGCACGGAGTTTGATTA
VP2162	hypothetical protein	TTGCCGCCAAGATTGTTTC
VPA0188	hypothetical protein	TTGGCATTTAAGTTGCTCT
**Predicted by the RpoN binding motif of** ***V. parahaemolyticus***		
VP2250	flagellar hook-basal body complex protein FliE	ATGGCACATAAATTGCTGT
VP2252	PAS domain-containing protein FlaL	TTGGTACGCTAATTGCTTA
VPA0264	flagellar basal-body rod protein FlgB2	ATGGCACGTATCTTGTTTG
VPA1548	lateral flagellin LafA	GTGGCAAGCGACCTGCCTC
VPA1550	flagellar distal capping protein LafB	TAGGCACGTATCTTGCGAT
VPA1027	hypothetical protein (*hcp2*)	AAGGAGCGTATTTAAAATG
VPA1044	hypothetical protein	TTGGCCGAGAAAATCTAAC

The RpoN-binding site in the promoter of *flgB* and *fliE* is shown in Figures 3D,E. Next, a mutant DNA probe was constructed by deleting the conserved binding site, and the EMSA results revealed that RpoN could not directly bind to the mutant DNA probe of the Δ*flgB* promoter (Figure 3F) and Δ*fliE* promoter (Figure 3G). Our results indicate that the RpoN protein can bind to the conserved −24 box and − 12 box in the promoters of *flgB* and *fliE* to regulate the expression of the polar flagellar gene clusters.

### RpoN directly binds to the promoters of *flgB2* and *lafA* to regulate the swarming motility of *V. parahaemolyticus*

**Figure 4:**
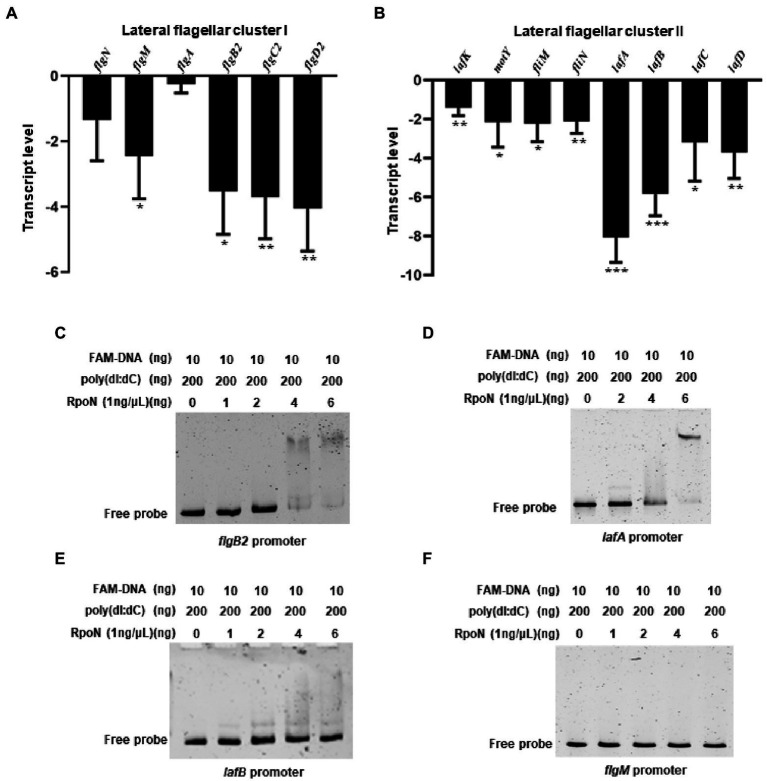
RpoN directly binds to the promoters of *flgB2* and *lafA* to regulate the expression of lateral flagellar genes in *V. parahaemolyticus*. **(A,B)** qRT-PCR analysis of the transcription levels of lateral flagellar cluster I **(A)** and lateral flagellar cluster II **(B)** genes in Δ*rpoN* compared to WT. The data are presented as the mean ± SD (*n* = 3). *p < 0.05, **p < 0.01, ***p < 0.001, Student’s *t* test analyzes Δ*rpoN* compared to WT. **(C–F)** EMSA analysis the specifically binds of RpoN to the promoters of *flgB2* **(C)**, *lafA* **(D)**, *lafB* **(E)**, and *flgM* **(F)**.

Previous studies have shown that the *V. parahaemolyticus* contains two flagellar systems (McCarter, 2004). However, RNA-seq of the bacteria cultured in LB medium could not identify the transcription level of genes responsible for the lateral flagellar system, which mediates bacterial swarming motility (McCarter, 2004). The swarming ability of the Δ*rpoN* strain was significantly lower than that of the WT and *rpoN*^+^ strain (Supplementary Figure S1B), which is consistent with the previously study (Whitaker et al., 2014). Our previous study also shown that the lateral flagellar systems contained two clusters, lateral flagellar cluster I and lateral flagellar cluster II (Gu et al., 2019). Then the qRT-CPR was used to verify the regulation of RpoN to lateral flagellar gene clusters, and the results showed that the expression of *flgM*, *flgB2C2D2*, *lafK*, *motY*, *fliMN*, *lafA*, and *lafBCD* genes were significantly down-regulated in the Δ*rpoN* strain compared to WT (Figures 4A,B). In addition, the RpoN-binding motif of *V. parahaemolyticus* were used to blast against the lateral flagellar gene clusters, and found that promoters of *flgB2*, *lafA*, and *lafB* contain the potential RpoN binding sites. Then, the above three promoters and the *flgM* promoter were selected to confirm by EMSA. The results showed that the RpoN protein could directly bound to the promoters of *flgB2* (Figure 4C) and *lafA* (Figure 4D), whereas it could not directly bound to the promoters of *lafB* (Figure 4E) and *flgM* (Figure 4F). These results indicated that the RpoN protein could directly bind to the promoters of *flgB2* and *lafA* to regulate the expression of lateral flagellar gene cluster and mediate the swarming motility of *V. parahaemolyticus*.

### RpoN directly regulates the expression of T6SS2 to mediate the adhesion of *V. parahaemolyticus*

**Figure 5:**
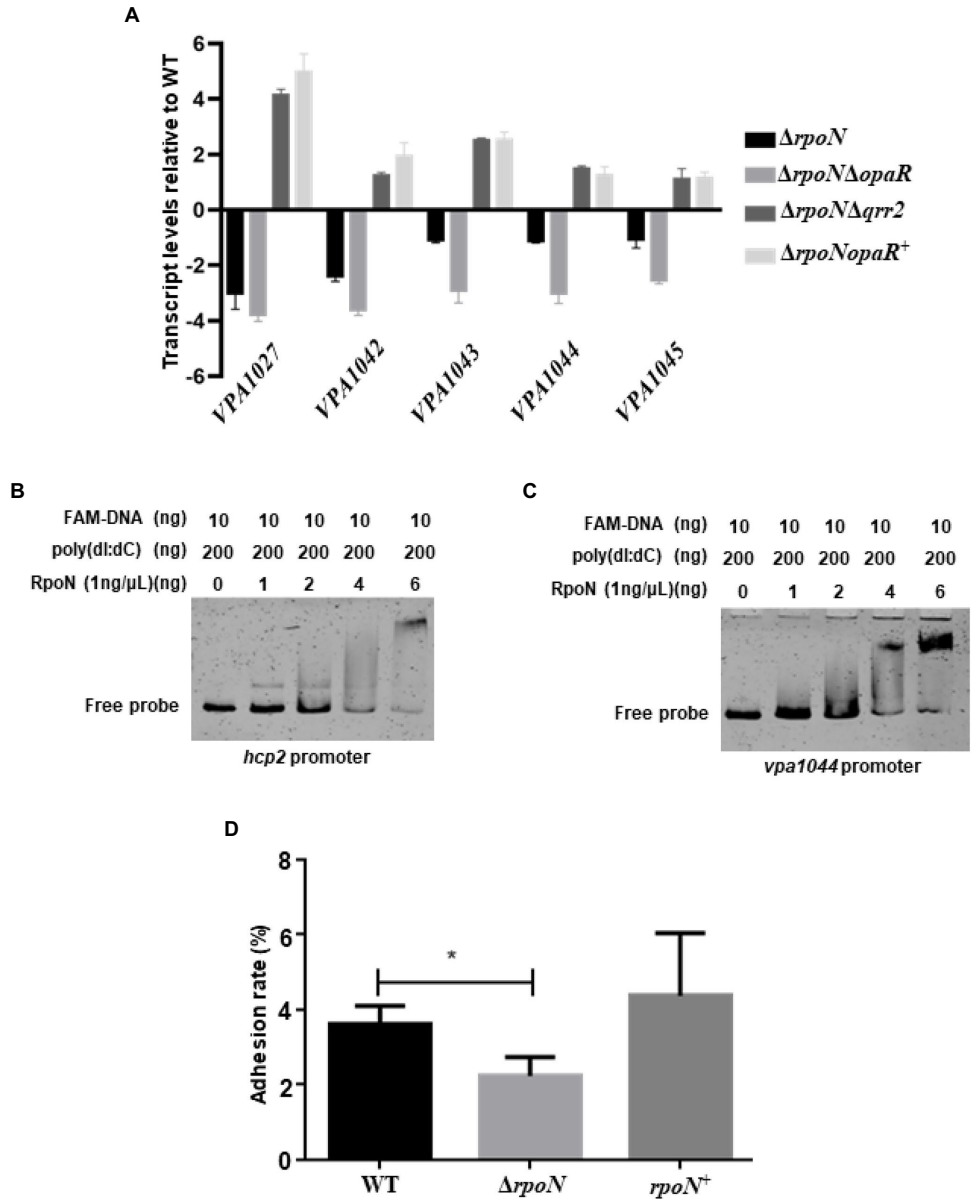
The RpoN directly regulates the expression of T6SS2 to mediate the adhesion of *V. parahaemolyticus* to HeLa cells. **(A)** qRT-PCR analysis of the expression levels of genes located in T6SS2 in WT, Δ*rpoN*, Δ*rpoN*Δ*opaR*, *ΔrpoNΔqrr2* and *ΔrpoNopaR^+^* strains. The data are presented as the mean ± SD (*n* = 3). Student’s *t* test analyzes the different mutant strains compared to WT, and all the *p-value* lower than 0.001. **(B,C)** EMSA analysis of the specific binding of RpoN protein to the promoters of *hcp2* **(B)** and *VPA1044* **(C)**. **(D)** Adhesion analysis of WT, Δ*rpoN*, and *rpoN^+^* strains to HeLa cell monolayers. The data are presented as the mean ± SD (*n* = 3), *p < 0.05, Student’s *t* test analyzes Δ*rpoN* compared to WT.

RNA-seq results also showed that the T6SS2 gene cluster was significantly down-regulated in the Δ*rpoN* strain compared to WT (Table 1). qRT-PCR results confirmed that RpoN could regulate the expression of T6SS2 genes (Figure 5A). EMSA results found that the RpoN protein could directly bind to the promoters of *hcp2* and *VPA1044* to regulate the expression of T6SS2 (Figures 5B,C). The previous study has revealed that the T6SS2 predominately contributes to the adhesion of *V. parahaemolyticus* to host cells (Yu et al., 2012). Therefore, the adhesion rates of WT and Δ*rpoN* strains to HeLa cells were also determined in this study. The results showed that the adhesion rate of the Δ*rpoN* strain was significantly lower than that of the WT strain, while the adhesion rate of the *rpoN*^+^ strain was restored to the same level as WT (Figure 5D). These results indicated that the RpoN protein could directly regulate the expression of T6SS2 to mediate the adhesion of *V. parahaemolyticus* to HeLa cells.

### RpoN regulates the expression of metabolic genes in *V. parahaemolyticus*

**Figure 6:**
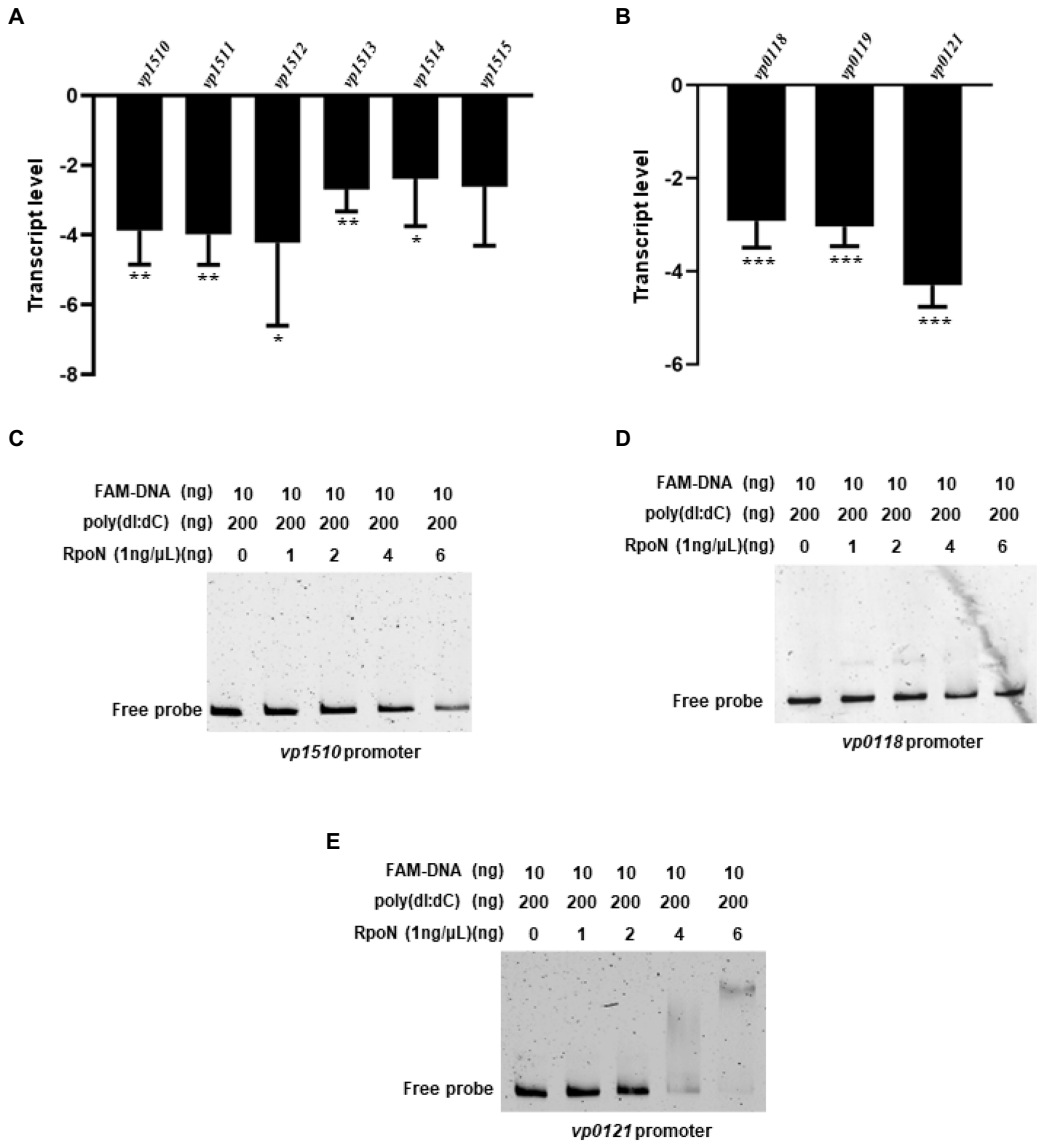
The RpoN regulates the expression of metabolism-associated genes. **(A)** qRT-PCR analysis of the expression levels of formate dehydrogenase genes (*VP1510*-*VP1515*) in Δ*rpoN* compared to WT. **(B)** qRT-PCR analysis of the expression levels of nitrogen regulatory protein (*VP0118-VP0119*) and glutamine synthetase (*VP0121*) associated genes in Δ*rpoN* compared to WT. The data are presented as the mean ± SD (*n* = 3). *p < 0.05, **p < 0.01, ***p < 0.001, Student’s *t* test analyzes Δ*rpoN* compared to WT. **(C–E)** EMSA analysis of the specific binding of RpoN protein to the promoters of *VP1510* **(C)** and *VP0118* **(D)** and VP0121 **(E)**.

RNA-seq analysis showed that the RpoN protein could regulate the expression of nitrogen regulation protein (*VP0118*), glutamine synthetase (*VP0121*), and the formate dehydrogenase gene cluster (*VP1510*-*VP1515*; Table 1). Then, the qRT-PCR also confirmed that the expression of these genes was down-regulated in the Δ*rpoN* strain compared to WT (Figures 6A,B). Furthermore, the *VP0121* and *VP1510* promoters contained the predicted RpoN binding sites (Table 2), whereas the *VP0118* promoter did not contain the RpoN binding sites. The EMSA results showed that the RpoN protein could not bind to the promoters of *VP1510* (Figure 6C) and *VP0118* (Figure 6D), but it could directly bind to the promoter of *VP0121* (Figure 6E). These results indicated that the RpoN directly bound to the promoter of *VP0121* to regulate the expression of the genes associated with glutamine synthetase, and indirectly regulate the expression of genes associated with nitrogen regulation and the formate dehydrogenase in *V. parahaemolyticus*.

### RpoN played essential roles in hemolytic activity and cytotoxicity.

**Figure 7:**
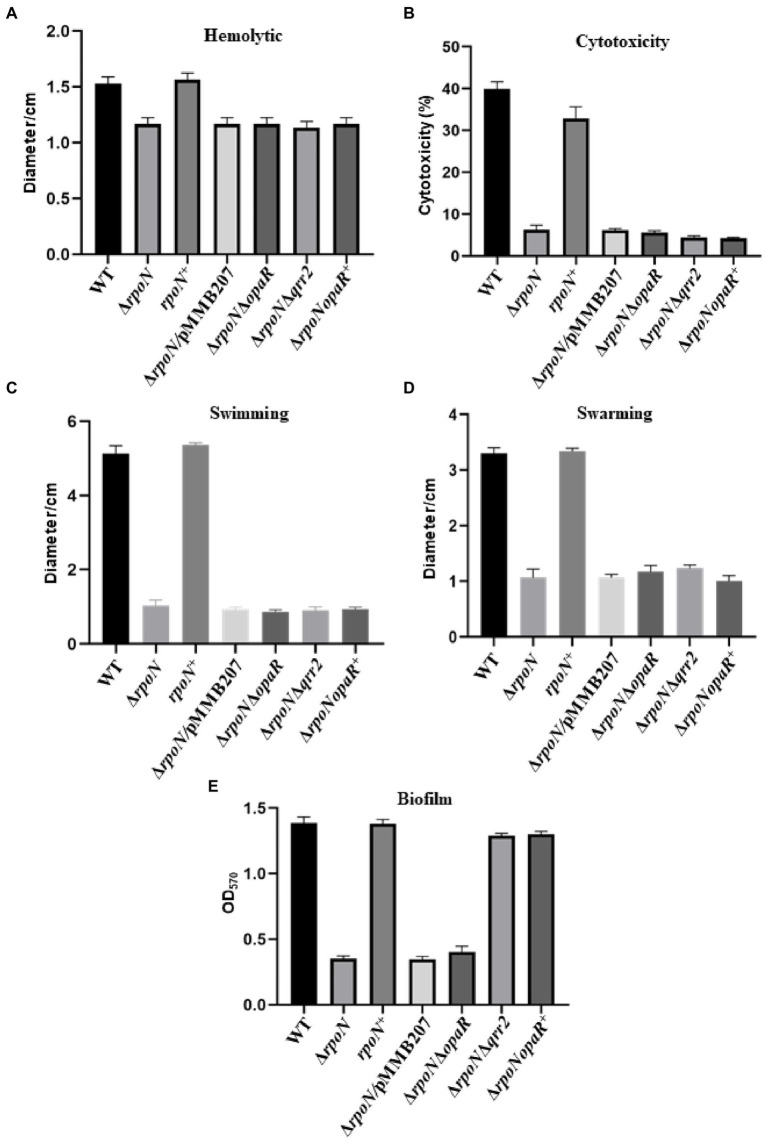
The RpoN regulates the phenotypes in an OpaR-dependent or OpaR-independent way. **(A)** Hemolytic activity assay of WT, ∆*rpoN*, *rpoN^+^*, ∆*rpoN*/pMMB207, Δ*rpoN*Δ*opaR*, *ΔrpoNΔqrr2* and *ΔrpoNopaR^+^* strains grown on sheep blood agar plates at 37°C. **(B)** Assessment of cytotoxicity of WT, ∆*rpoN*, *rpoN^+^*, ∆*rpoN*/pMMB207, Δ*rpoN*Δ*opaR*, *ΔrpoNΔqrr2* and *ΔrpoNopaR^+^* strains towards HeLa cell monolayers. **(C,D)** Analyze of swimming **(C)** and swarming **(D)** of WT, ∆*rpoN*, *rpoN^+^*, ∆*rpoN*/pMMB207, Δ*rpoN*Δ*opaR*, *ΔrpoNΔqrr2* and *ΔrpoNopaR^+^* strains in LB medium or BHI medium, respectively. **(E)** Biofilm formation of WT, ∆*rpoN*, *rpoN^+^*, ∆*rpoN*/pMMB207, Δ*rpoN*Δ*opaR*, *ΔrpoNΔqrr2* and *ΔrpoNopaR^+^* strains. The data are presented as the mean ± SD (*n* = 3), Student’s *t* test analyzes different mutant strains compared to WT, and all the *p-value* lower than 0.001.

In addition, our results also showed that the hemolytic activity of the Δ*rpoN* strain was significantly lower than that of the WT and the complemented strain (Figure 7A). To further assess the function of RpoN in the host cell, the cytotoxicity of the WT and Δ*rpoN* mutant strains toward HeLa cells was evaluated. The cytotoxicity of the Δ*rpoN* strain was significantly lower than that of the WT and the *rpoN*^+^ strain (Figure 7B). These results suggest that RpoN plays an essential role in regulating hemolytic activity, and cytotoxicity toward HeLa cells.

### Role of OpaR in the regulation of RpoN to virulence-associated phenotypes

Our RNA-seq results showed that the RpoN protein could regulate the expression of *opaR* and was confirmed by qRT-PCR (Supplementary Figure S4A), and EMSA results showed that RpoN protein could not directly bound to the promoter of *opaR* (Supplementary Figure S4B). A previous study showed that the regulation of RpoN to *opaR* was dependent on the *qrr2* (Tague et al., 2022). Thus, we constructed the Δ*rpoN*Δ*opaR*, *ΔrpoNΔqrr2*, and *ΔrpoNopaR^+^* strains to investigate whether the regulation of RpoN to hemolytic, cytotoxicity, and motility was through OpaR. The expression levels of *opaR* in these strains were shown in Supplementary Figure S4A. The hemolytic activity of Δ*rpoN*Δ*opaR*, *ΔrpoNΔqrr2*, and *ΔrpoNopaR^+^* strains were significantly lower than that in WT, and similar to the Δ*rpoN* (Figure 7A), indicating that regulation of RpoN to hemolytic was not associated with OpaR. The similar results were found in cytotoxicity and motility (Figures 7B–D). The above results indicated that RpoN regulates hemolytic activity, cytotoxicity, and motility of *Vibrio parahaemolyticus* without a relationship to OpaR.

Next, we also investigate whether the regulation of RpoN to T6SS2 and biofilm was through OpaR. As shown in the Figure 5A, the expression of T6SS2 genes (*VPA1027*, *VPA1042*, *VPA1043*, *VPA1044*, and *VPA1045*) were significantly increased in the *ΔrpoNΔqrr2* and *ΔrpoNopaR^+^* strains compare to WT, indicating that the regulation of RpoN to T6SS2 was related to OpaR. Furthermore, the biofilm formation was decreased in the Δ*rpoN* and Δ*rpoN*Δ*opaR* strains, while it was restored to the level of WT in *ΔrpoNΔqrr2* and *ΔrpoNopaR^+^* strains (Figure 7E). These results indicated that the regulation of RpoN to T6SS2 and biofilm formation of *Vibrio parahaemolyticus* with a close relationship to OpaR.

## Discussion

In *V. parahaemolyticus*, RpoN has been reported to regulate carbon utilization and affect host colonization (Whitaker et al., 2014). In other bacteria, RpoN protein also has been reported to regulate the expression of virulence-associated genes, such as those associated with flagellar systems, biofilm formation, QS, and T6SS (Zhao et al., 2010; Dong and Mekalanos, 2012; Shao et al., 2018; Liu et al., 2021b). The present study used RNA-seq to investigate the genes regulated by the alternative sigma factor RpoN in *V. parahaemolyticus*. RNA-seq results revealed that the expression of 399 genes significantly differed between the Δ*rpoN* and WT strains. Moreover, 264 genes were positively regulated by RpoN, including those associated with carbon utilization, QS, flagellar systems, and T6SS (Figure 1). Similarly, 70 genes were positively regulated by RpoN in *Escherichia coli* K-12, including genes associated with bacterial motility and nitrogen metabolism (Zhao et al., 2010). In *V. cholerae*, 144 genes have been reported to be positively regulated by RpoN, including those associated with motility, T6SS, nitrogen utilization, and phage shock protein synthesis (Dong and Mekalanos, 2012). In *P. aeruginosa*, the genes taking up approximately 20% of the genome is regulated by RpoN (Damron et al., 2012). The current observations suggest that the sigma factor RpoN plays an essential role in various biological processes in different bacteria and diverse hosts. Similar studies have been conducted in the *V. cholerae* and *E. coli* (Zhao et al., 2010; Dong and Mekalanos, 2012). In addition to defining the RpoN regulon in *V. parahaemolyticus*, we also identified two new RpoN-binding promoters (*flgB*2 and *lafA*) and, for the first time, showed that RpoN plays an essential role in hemolytic activity, adhesion, and cytotoxicity. These results provide additional insight into the potential role of RpoN in pathogenesis.

*V. parahaemolyticus* contains two distinct flagellar systems for expression under different circumstances and facilitates the infection process (Merrell et al., 1984; McCarter, 2004). Compared with the WT strain, swimming motility and swarming motility were defective in the Δ*rpoN* strain in the previous study (Whitaker et al., 2014). Furthermore, our qRT-PCR data confirmed that RpoN could regulate the expression of the polar flagellar clusters and lateral flagellar clusters to mediate motility in *V. parahaemolyticus* (Figures 2, 4). The previous study has demonstrated that RpoN directly regulates the expression of flagellar systems in *V. cholerae* (Dong and Mekalanos, 2012). Based on the conserved binding motif of RpoN in *V. cholerae*, we searched for the potential RpoN-binding sites in the promoters of flagellar gene clusters in *V. parahaemolyticus*. Our results revealed that the promoters of *flgB*, *flgF*, *flgK*, *flhA*, *fliE*, and *flaL* in the polar gene clusters contained RpoN-binding sites, and the promoters of lateral flagellar genes *flgB2*, *lafA* and *lafB* contained the conserved RpoN-binding sites (Table 2). Nevertheless, the EMSA results revealed that the RpoN protein directly bind to the promoters of *flgB*, *fliE*, *flgB2*, and *lafA* (Figures 3B,C, 4C,D). Notably, a previous study reported that *flaK* is a σ^54^-dependent regulator of polar flagellar Class II genes in *V. parahaemolyticus* (McCarter, 2004). In addition, *flgB*, *flgF*, *flgA*, *flgK*, *fliD*, *fliE*, *fliK*, *flhA*, *motY*, and *flgT* have been reported to be RpoN-binding promoters in *V. cholerae* (Dong and Mekalanos, 2012). Therefore, we confirmed that RpoN could bind to the promoters of *flgB* and *fliE* in polar flagellar gene clusters by EMSA, and identified two new RpoN-binding promoters of *flgB*2 and *lafA* in lateral flagellar gene clusters.

Two T6SS loci (T6SS1 and T6SS2) have been identified in *V. parahaemolyticus*. T6SSs are tightly regulated by transcription regulators and sigma factors (Wettstadt, 2020; Yin et al., 2020; Shao et al., 2021). H-NS, QS, ToxR, TfoY, QsvR, and CalR have been identified as regulators to control the expression or secretion of T6SS in *V. parahaemolyticus* (Zhang et al., 2017a,b; Qiu et al., 2020). RpoN was also detected to directly control the expression of *hcp* but not that of the major cluster of T6SS in *V. cholerae* (Dong and Mekalanos, 2012). However, our RNA-seq data and qRT-PCR analysis revealed that the T6SS2 genes were down-regulated in the Δ*rpoN* strain compared to the WT strain (Table 1; Figure 5A). EMSA results also indicated that the RpoN protein could directly bind to the promoters of *hcp2* and *VPA1044* (Figures 5B,C). However, RpoN-binding sites in the promoters of *hcp2* and *VPA1044* were incomplete, containing only the conserved −24 box (GG) but without the conserved −12 box (Table 2). Furthermore, it was reported that RpoN could directly bind to the promoters of *hcpA* and *hcpB* to positively control T6SS expression in *P. aeruginosa*, which also only contain the conserved −24 box (GC; Shao et al., 2018). The −24 element is an attachment determinant for RpoN, whereas the −12 element is variable (Wang and Gralla, 1988; Barrios et al., 1999), which may explain the RpoN bound to the less conserved binding sites in the promoters of *hcp2* and *VPA1044*. Therefore, our results indicated that RpoN protein not only directly controls the expression of *hcp2*, but also directly binds to the promoter of *VPA1044* to regulate the transcription of T6SS2 gene cluster in *V. parahaemolyticus*.

In *V. cholerae*, RpoN was identified directly regulate the metabolism-associated genes responsible for nitrogen utilization and formate dehydrogenase by ChIP-seq (Dong and Mekalanos, 2012). Our RNA-seq results revealed that the genes associated with nitrogen regulatory protein (*VP0118*), glutamine synthetase (*VP0121*), and formate dehydrogenase (*VP1510*-*VP1515*) were significantly down-regulated in the Δ*rpoN* strain in comparison with the WT strain (Table 1). BLAST analysis revealed the presence of RpoN-binding sites in the promoters of *VP0118*, *VP0121*, and *VP1510* (Table 2). Thus, we speculated that RpoN could directly regulate the expression of these genes in *V. parahaemolyticus*. The EMSA results indicated that the RpoN protein could bind to the promoter of *VP0121* for glutamine synthetase (Figure 6E), and *V. parahaemolyticus* RpoN mutant could not grow in M9G medium containing ammonium as a sole nitrogen source (Whitaker et al., 2014). Our results confirmed that the RpoN protein could directly bind to the promoter of *VP0121* and regulate its expression for nitrogen utilization in *V. parahaemolyticus*. Besides, no binding shift was found in the promoters of *VP0118* and *VP1510* with the highest concentration of RpoN protein (Figures 6C,D). The previous studies demonstrated that co-factors could be necessary for the protein to bind the promoters *in vitro* (Bitoun et al., 2012). A previous study used the σ^54^-RNA polymerase complex to analyze the binding of RpoN with promoters (Berbard et al., 2011). These may explain why RpoN protein could not bind the promoters containing the conserved binding sites. In addition, there may be an indirect regulation event. A previous study has shown that the deletion of *rpoN* results in changes in the expression of genes that are directly controlled by other sigma factors (Dong et al., 2011). For example, the deletion of *rpoN* increases the expression of genes controlled by another sigma factor RpoS in *E. coli* (Dong et al., 2011). In our RNA-seq data, another sigma factor VP2210 was significantly up-regulated in the Δ*rpoN* strain compared to WT. VP2210 is known to directly regulate the expression of *exsC* and positively regulate the expression of T3SS1 (Gu et al., 2020). These data would suggest that RopN indirectly controls T3SS1 gene expression *via* down-regulating VP2210.

Moreover, the QS high cell density master regulator OpaR and sensor protein LuxN were also positively regulated by RpoN in *V. parahaemolyticus* (Table 1; Supplementary Figure S3). The RpoN protein could directly bind to the *lasI* promoter and positively regulate the *las*-QS system in *P. aeruginosa* (Shao et al., 2018). On the contrary, RpoN together with LuxO could activate the small regulatory RNA to negatively control the transcription of *hapR* at low cell density (LCD) in *V. cholerae* (Cheng et al., 2018). In *V. alginolyticus*, RpoN protein could positively regulate the transcription of *luxR* at LCD, whereas it could negatively regulate the transcription of *luxR* at high cell density (HCD; Zhang et al., 2021b). In *V. parahaemolyticus*, the expression of *opaR* was off at LCD and on at HCD, and the sRNA *qrr2* could inhibit the expression of *opaR* (Gode-Potratz and McCarter, 2011). In this study, the RNA-seq and qRT-PCR results showed that RpoN could positively regulate the expression of *opaR* dependent on the *qrr2* in *V. parahaemolyticus*. The above studies indicate that the regulation of QS by RpoN differs in different pathogens. In addition, RpoN can regulate virulence-associated genes responsible for motility, biofilm formation, T6SS, and host colonization in *V. cholerae*, *P. aeruginosa*, and *C. jejuni* (Dong and Mekalanos, 2012; Shao et al., 2018; Sher et al., 2020). Our RNA-seq data and qRT-PCR results also revealed that RpoN could positively regulate the expression of flagellar systems, T6SS2 and metabolic-associated genes (Table 1; Figures 2–6). Above all, our results further supporting that RpoN plays a global regulatory role in metabolic and virulence-associated pathways in *V. parahaemolyticus*.

Our results shown that RpoN can regulate the expression of virulence-associated genes responsible for hemolytic activity, cytotoxicity, motility, biofilm formation, and T6SS2, and most of these phenotypes are found to be controlled by OpaR (Lu et al., 2019, 2021; Tague et al., 2022; Wu et al., 2022). OpaR is a master regulator of quorum sensing that is known to directly regulate the expression of polar and lateral flagellar genes to inhibit swimming and swarming motility (Lu et al., 2019; Lu et al., 2021). Our results also showed that the RpoN could directly bound to the promoters of *flgB*, *fliE*, *flgB*2, and *lafA* to induce the expression of flagellar genes (Figures 3, 4). The expression of OpaR was decreased in the Δ*rpoN*Δ*opaR* compared to Δ*rpoN*, but the swimming and swarming motility was no different in these strains, indicating that both RpoN and OpaR could regulate the motility and RpoN plays a more important role in motility. Furthermore, both OpaR and RpoN could directly bind to *hcp2* and *VPA1044* promoters to positively regulate the expression of T6SS2 (Wu et al., 2022). Our results further found that the expression of T6SS2 genes in Δ*rpoN*Δ*opaR* was lower than that in Δ*rpoN*, whereas the expression of these genes was increased in the *ΔrpoNΔqrr2* and *ΔrpoNopaR^+^* strains (Figure 5A), indicating that both RpoN and OpaR could directly regulate the expression of T6SS2, and RpoN regulate T6SS2 with a close relationship to OpaR.

## Conclusion

**Figure 8:**
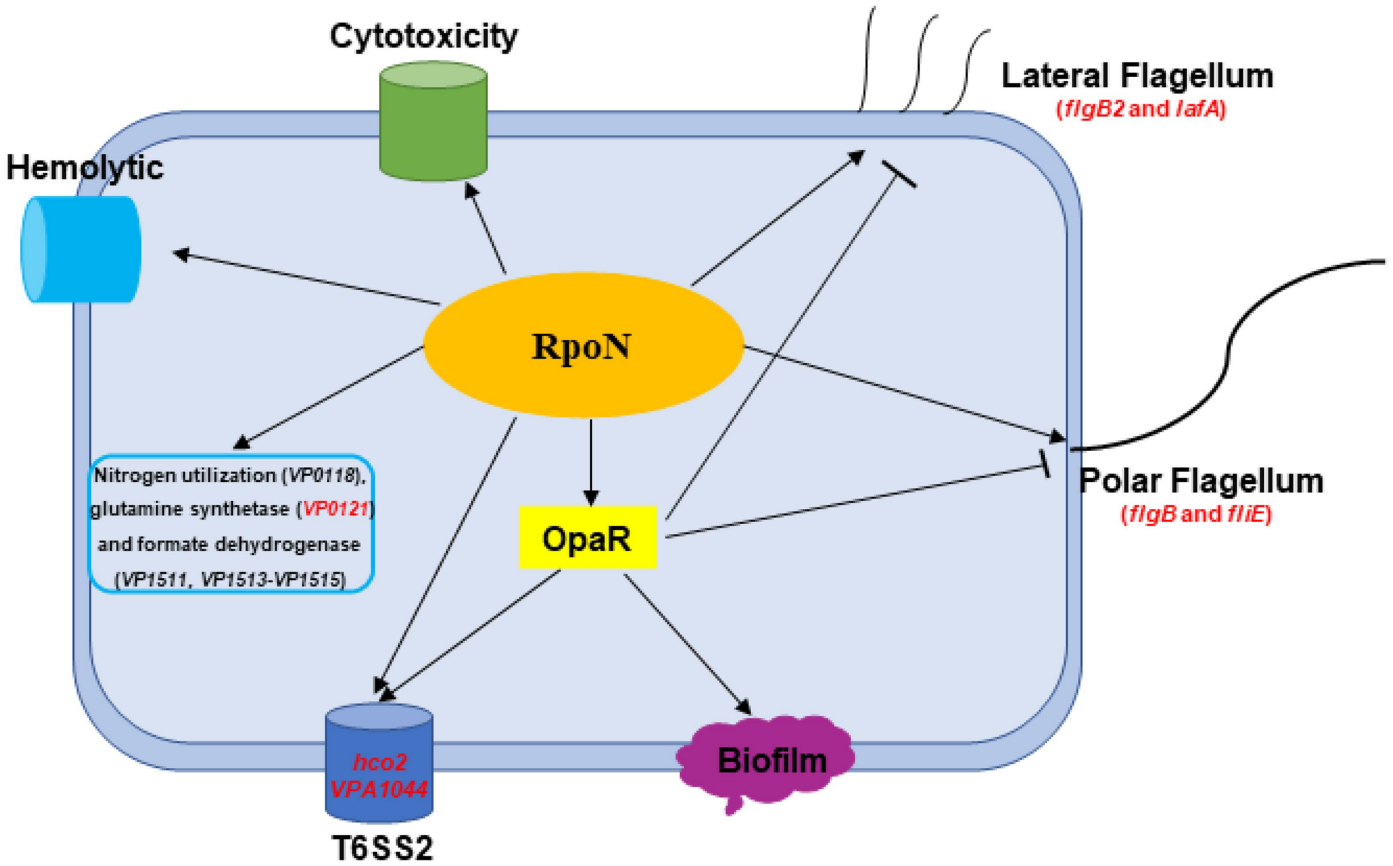
A schematic of the global regulatory networks of RpoN in *V. parahaemolyticus*. The RpoN directly binds to promoter regions of *flgB*, *fliE*, *flgB2*, and *lafA*, and positively regulates swimming and swarming motility in *V. parahaemolyticus*. RpoN protein also directly bind to promoters of *hcp2* and *VPA1044* to regulate the expression of T6SS2. Furthermore, RpoN protein could directly regulate the expression of *VP0121* to be responsible for nitrogen utilization. In addition, RpoN regulates the hemolytic activity, cytotoxicity, and motility in an OpaR-independent way, whereas RpoN regulates the T6SS2 and biofilm in an OpaR-dependent way in *Vibrio parahaemolyticus*. The red genes indicated the promoters directly bound by RpoN protein. The arrows indicate activation, while the bar-ended lines indicate repression.

In this study, Figure 8 shown the RpoN-controlled virulence pathways in the flagellar systems, biofilm formation, T6SS2, hemolytic, cytotoxicity, and QS systems in *V. parahaemolyticus*. Besides, RpoN could regulate the metabolism pathway, including nitrogen regulatory protein, glutamine synthetase, and formate dehydrogenase. Furthermore, RpoN displayed direct binding to the promoters and controlled the expression of *flgB, fliE*, *flgB2*, *lafA*, *hcp2*, *VPA1044*, and *VP0121*, and mediated the motility, T6SS2, and glutamine utilization in *V. parahaemolyticus*. In addition, RpoN also contributed to the hemolytic activity, adhesion, and cytotoxicity of *V. parahaemolyticus*. Thus, our study suggests that RpoN is a global regulator that controls a large group of metabolic and virulence-associated pathways in *V. parahaemolyticus*, further supporting the conserved function of RpoN in many bacteria.

## Data availability statement

The datasets presented in this study can be found in online repositories. The names of the repository/repositories and accession number(s) can be found in the article/Supplementary material.

## Author contributions

DG, YZ, and KW contributed to the conception, design and performed the experiments. DG, YZ, KW, and ML were responsible for the acquisition of the data analyzed in this study. DG, KW, and XJ were involved in the analysis and interpretation associated with this work. All authors contributed to the article and approved the submitted version.

## Funding

This work was supported by the National Natural Science Foundation of China (32070127); the Priority Academic Program Development of Jiangsu Higher Education Institutions (PAPD).

## Conflict of interest

The authors declare that the research was conducted in the absence of any commercial or financial relationships that could be construed as a potential conflict of interest.

## Publisher’s note

All claims expressed in this article are solely those of the authors and do not necessarily represent those of their affiliated organizations, or those of the publisher, the editors and the reviewers. Any product that may be evaluated in this article, or claim that may be made by its manufacturer, is not guaranteed or endorsed by the publisher.

## Footnotes

^1^ http://meme-suite.org
