# Supplementary material for: Characterization of the RpoN regulon reveals the regulation of motility, T6SS2 and metabolism in Vibrio parahaemolyticus
Source: Front Microbiol. 2022 Dec 22;13:1025960. doi: 10.3389/fmicb.2022.1025960 (PMC9817140; doi:10.3389/fmicb.2022.1025960)
Supplement: Supplementary file 1 [file Data_Sheet_1.pdf]

Supplementary materials:

Table S1. Bacterial strains and plasmids used in this study.

Table S2. Primers used in this study.

Figure S1. RpoN regulates swimming and swarming motility in *V. parahaemolyticus*. (A) Swimming motility analysis of WT, Δ*rpoN*, *rpoN*$^{+}$, and Δ*rpoN*/pMMB207 cultures on LB plates with 0.3% agar at 37°C. (B) Swarming motility assay of WT, Δ*rpoN*, *rpoN*$^{+}$, and Δ*rpoN*/pMMB207 strains grown on a BHI agar plate with 1.5% agar at 30°C. IPTG (1 mM) was used to induce the expression of RpoN in *rpoN*$^{+}$ and Δ*rpoN*/pMMB207 cultures. The data are presented as the mean ± SD (n = 3). ****p < 0.0001, Student's *t* test.

Figure S2. The RpoN cannot directly bind to the promoters of *flgF*, *flgK*, *flaL*, *flhA* and *gyrB*. EMSA was performed to assess the binding of RpoN to the promoter regions of *flgF* (A), *flgK* (B), *flaL* (C), *flhA* (D) *and gyrB* (E).

Figure S3. RpoN positively regulates the expression of *opaR*. qRT-PCR analysis of the transcription levels of *opaR* in Δ*rpoN* compared to WT. The data are presented as the mean ± SD (n = 3). ***p < 0.001, Student's *t* test. (**B**) EMSA was performed to assess the binding of RpoN to the promoter of *opaR*.

Figure S4. Growth curve of *V. parahaemolyticus* WT and *rpoN* mutant strains. The WT, Δ*rpoN*, *rpoN*$^{+}$ and Δ*rpoN*/pMMB207 strains were grown at 37°C in LB medium with 1% NaCl, and measured the value of OD_{600} at the indicated time.

Table S1. Bacterial strains and plasmids used in this study

Strains or plasmids	Relevant characteristics	Reference
E. coli		
DH5α λ*pir*	Host for π requiring plasmids	(Wang et al., 2015)
SM10 λ*pir*	Host for π requiring plasmids, conjugal donor	(Liang et al., 2003)
BL21(DE3)	Host strain for protein expression	Tiangen
BL21/pET32a::*rpoN*	BL21, pET32a carrying the *rpoN* ORF, Carb^{r}	This study
V. Parahemolyticus		
RIMD2210633	Wild type, O3:K6 clinical isolate, Carb^{r}	(Makino et al., 2003)
Δ*rpoN*	RIMD2210633, in-frame deletion in *rpoN*, Amp^{r} or Carb^{r}	This study
rpoN^{+}	Δ*rpoN* complemented strain harboring *rpoN*::pMMB207, Carb^{r}, Cm^{r}	This study
Δ*rpoN*/pMMB207	Δ*rpoN* strain harboring pMMB207 , Carb^{r}, Cm^{r}	This study
**Plasmids**		
pDM4	Suicide vector, λ*pir* dependent, R6K, *SacBR*, Cm^{r}	(Milton et al., 1996)
pMMB207	Expressing vector, Cm^{r}	(Morales et al., 1991)
pET32a	IPTG induced expressing vector, Carb^{r}	Novagen
pDM4::Δ*rpoN*	Up and downstream of *rpoN* insert into pDM4, Cm^{r}	This study
pMMB207::*rpoN*	RBS and *rpoN* ORF insert into pMMB207, Cm^{r}	This study
pET32a::*rpoN*	*rpoN* ORF insert into pET32a, Carb^{r}	This study

REFERENCE:

Liang WL, Wang SX, Yu FG, Zhang LJ, Qi GM, Liu YQ, Gao SY, Kan B. (2003). Construction and evaluation of a safe, live, oral *Vibrio cholerae* vaccine candidate, IEM108. *Infect. Immun.* 71, 5498-5504. doi: 10.1128/IAI.71.10.5498-5504.2003.

Makino K, Oshima K, Kurokawa K, Yokoyama K, Uda T, Tagomori K, Iijima Y, Najima M, Nakano M, Yamashita A, Kubota Y, Kimura S, Yasunaga T, Honda T, Shinagawa H, Hattori M, Iida T. (2003). Genome sequence of *Vibrio parahaemolyticus*: a pathogenic mechanism distinct from that of *V cholerae*. *Lancet*. 361, 743-749. doi: 10.1016/S0140-6736(03)12659-1.

Milton DL, O'Toole R, Horstedt P, Wolf-Watz H. (1996). Flagellin A is essential for the virulence of *Vibrio anguillarum*. *J. Bacteriol.* 178, 1310-1319. doi:10.1128/jb.178.5.1310-1319.1996.

Morales VM, Bäckman A, Bagdasarian M. (1991). A series of wide-host range low-copy-number vectors that allow direct screening for recombinants. *Gene*. 97, 39-47. doi: 10.1016/0378-1119(91)90007-x.

Wang Q, Millet YA, Chao MC, Sasabe J, Davis BM, Waldor MK. (2015). A genome-wide screen reveals that the *Vibrio cholerae* phosphoenolpyruvate phosphotransferase system modulates virulence gene expression. *Infect. Immun.* 83, 3381-3395. doi: 10.1128/IAI.00411-15.

Table S2. Primers used in this study

Primer name	Primer sequence (5’ to 3’)	Target
rpoN up-F	GAGCGGATAACAATTTGTGGAATCCCGGGACATTGACGACAAAGACATCAGTATT	For *rpoN* mutant
rpoN up-R	TAGTGCCTTACATTCAGTGTTACTTACCTTGTAAT	For *rpoN* mutant
rpoN down-F	AACACTGAATGTAAGGCACTAACTGAAAAGGAAAGT	For *rpoN* mutant
rpoN down-R	AGCGGAGTGTATATCAAGCTTATCGATACCACTCAAACAGCTCGGTAGAATCTTG	For *rpoN* mutant
rpoN out-F	TATTGGAAACTCGCCAAGAAATGAC	For *rpoN* mutant
rpoN out-R	TCAAATTCAATTGCTTCATCGCATT	For *rpoN* mutant
rpoN in-F	GTTAGACGATGATATGCCGGTCTAC	For *rpoN* mutant
rpoN in-R	CCAAGGATTCTCGATATTTAGCTAT	For *rpoN* mutant
opaR-up-F	TGTGGAATCCCGGGAGAGCTCATAGATTTAGGTTCACGTAGCGATAGG	For *opaR* mutant
opaR-up-R	GAGCTATCCATTTTCCTTGCCATTTGA	For *opaR* mutant
opaR-down-F	GCAAGGAAAATGGATAGCTCAGATTTGAACACGAACAAG	For *opaR* mutant
opaR-down-R	AAGCTTATCGATACCGTCGACTGCTCACAAAGGTGTGCATGA	For *opaR* mutant
opaR-out-F	ATCAACTGGCACATTTACTTCAC	For *opaR* mutant
opaR-out-R	TTTACACCAAGCGTATCAAAGAC	For *opaR* mutant
opaR-in-F	ATCAACTGGCACATTTACTTCAC	For *opaR* mutant
opaR-in-R	TGCTTGTACGAACAGCGAGTAA	For *opaR* mutant
Qrr2-up-F	TGTGGAATCCCGGGAGAGCTCGCGCTCAACAACTAATGAAAGACA	For *Qrr2* mutant
Qrr2-up-R	AAAGCACTTACGTGCTTTGCAAATTGC	For *Qrr2* mutant
Qrr2-down-F	GCAAAGCACGTAAGTGCTTTATTTTTGGGCACAAGATC	For *Qrr2* mutant
Qrr2-down-R	AAGCTTATCGATACCGTCGACGACGGGTTAATACGACGAATGC	For *Qrr2* mutant
Qrr2-out-F	TTGATGGCGCTACGATTGGT	For *Qrr2* mutant
Qrr2-out-R	CGCTAAGGTTGCAATGCTCG	For *Qrr2* mutant
pDM4-F	CAGCAACTTAAATAGCCTCTAAGGT	For *rpoN* mutant
pDM4-R	GGTGCTCCAGTGGCTTCTGTTTCTA	For *rpoN* mutant
rpoN com-F	TCGGTACCCGGGGATCCTCTAGTAAGGAGGTAGGATAATAATGAAACCTTCATTACAACTCAAGC	For *rpoN* complement
rpoN com-R	TCCGCCAAAACAGCCAAGCTTTAAAGTAGGCGTTTACGCTGACTT	For *rpoN* complement
opaR-com-F	TCGGTACCCGGGGATCCTCTAGTAAGGAGGTAGGATAATAATGGACTCAATTGCAAAGAGACCTA	For *opaR* complement
opaR-com-R	TCCGCCAAAACAGCCAAGCTTTAGTGATGATGATGATGATGGTGTTCGCGATTGTAGATGCAA	For *opaR* complement
pMMB207-F	GAGCGGATAACAATTTCACACAGG	For *rpoN* complement
pMMB207-R	GATTTAATCTGTATCAGG	For *rpoN* complement
RpoN-F	CGACGACAAGGCCATGGCTGATATCATGAAACCTTCATTACAACTCAAGC	For RpoN Protein
RpoN-R	GATCTCAGTGGTGGTGGTGGTGGTGAAGTAGGCGTTTACGCTGACTT	For RpoN Protein
pET32a-F	TAATACGACTCACTATAGGG	For RpoN Protein
pET32a-R	GCTAGTTATTGCTCAGCGG	For RpoN Protein
fliE-F	TGCCTGCAGGTCGACGATGTGGAATGTAATGGCCGACGCAAAG	For EMSA
fliE-R	TATCACCTCTGAGCCAAAAGTTTGA	For EMSA

fliE-F1	CTTGCCGAGCGTCACTGTAGTAAAA	For EMSA
fliE-R1	AATTATTGACAAGACGCCAGCTGTATAGTTTAC	For EMSA
fliE-F2	CTGGCGTCTTGTCAATAATTAGTTTAAATAGA	For EMSA
fliE-R2	CACTGGCATGTTCATGAGGTCTTTG	For EMSA
flgB-F	TGCCTGCAGGTCGACGATTTTGTCTATATGAGATGAGATTTCG	For EMSA
flgB-R	GTGCCTTGTCAAAAGATATAGCCAT	For EMSA
flgB-F1	CGTAATGTGCTGATTTACTTCTCTC	For EMSA
flgB-R1	AATAACTAAAAAACCGGAAACGCTAAAACACC	For EMSA
flgB-F2	TTTCCGGTTTTTTAGTTATTAATAACGGTCAG	For EMSA
flgB-R2	TTTGATTGCCTTGGTTAAGTTCTTG	For EMSA
gyrB-F	TGCCTGCAGGTCGACGATTGTCCTAGAAGCGATTTATTTACTC	For EMSA
gyrB-R	TTTTGGACCATCCGTCAGTAAATCA	For EMSA
flgF-F	TGCCTGCAGGTCGACGATCTTGGCTTGATAACATCATGACATT	For EMSA
flgF-R	TTACTCCAAAAAAGTCTCTGTACTT	For EMSA
flgK-F	TGCCTGCAGGTCGACGATCTGCAAGTGAAGCAGAAAATCGAAA	For EMSA
flgK-R	CAGAAGATCTGACGCCATACCTGCC	For EMSA
flaL-F	TGCCTGCAGGTCGACGATGAGCTGCTGATATGCTGGGTATGCG	For EMSA
flaL-R	TCACTGCCTGTCGAAATATCGCCAC	For EMSA
flhA-F	TGCCTGCAGGTCGACGATCGCCGGAACATCGTCATTAATCGTA	For EMSA
flhA-R	ACTGCGAGGTATCTCAATCGAATTG	For EMSA
flgM-F	TGCCTGCAGGTCGACGATTTTTGGACTCAACGTAACATTT	For EMSA
flgM-R	GCTGTGGTCCTCATCATCATGT	For EMSA
flgB2-F	TGCCTGCAGGTCGACGATGTCCTGTCGAAAGAATTCCTA	For EMSA
flgB2-R	CTGGATGTACACCTAAAGCAT	For EMSA
lafA-F	TGCCTGCAGGTCGACGATTCTCTTTGCCTTAATCTTATGC	For EMSA
lafA-R	CTTAGTCTCCTTAGTTTATCAC	For EMSA
lafB-F	TGCCTGCAGGTCGACGATTTAATCAACACGTCACATTCCT	For EMSA
lafB-R	GAGCTTACTCCCTCTCTCATTG	For EMSA
hcp2-F	TGCCTGCAGGTCGACGATTCTGTAAATAATAATTGAGAGT	For EMSA
hcp2-R	GCTAATCTCCTAGAGCATTATTA	For EMSA
VPA1044-F	TGCCTGCAGGTCGACGATAAGGACTCCATATAAATGATGGG	For EMSA
VPA1044-R	GAAAAACCTCGCCAAAGACCTG	For EMSA
VP1510-F	TGCCTGCAGGTCGACGATGATATTTTATTGGCATGACATT	For EMSA
VP1510-R	GCTCAATTCCATTTATGAACA	For EMSA
VP0118-F	TGCCTGCAGGTCGACGATCCTTGTTTTATCCCATGGTCAGC	For EMSA
VP0118-R	GTACTAATCCTTGCAGCAAAAAC	For EMSA
VP0121-F	TGCCTGCAGGTCGACGATATCTTAATTCGCACCACTTTAG	For EMSA
VP0121-R	CTTGGATAACCTCCAGTGTTAA	For EMSA
FAM	TGCCTGCAGGTCGACGAT	For EMSA
flgB-RT-F	TGGGGGACAAACTGTATCGT	For qRT-PCR
flgB-RT-R	CCCTTTGATTGCCTTGGTTA	For qRT-PCR
flgC-RT-F	GCGTCATGCTGTATTTGGTG	For qRT-PCR
flgC-RT-R	AACCTGCACATTCGTTTGGT	For qRT-PCR
flgD-RT-F	TTTGAGGTAGGTGCGAAACC	For qRT-PCR

flgD-RT-R	CGCTGTTTACGTTGGCATAA	For qRT-PCR
flgE-RT-F	CAATGGATTTGCGTGTATCG	For qRT-PCR
flgE-RT-R	TGATTGGTTGTGGCTCGTAA	For qRT-PCR
flgF-RT-F	TCGCCATAAAACTCCAAACC	For qRT-PCR
flgF-RT-R	ATCTCTTCAGCGGTGCTCAT	For qRT-PCR
flgG-RT-F	GTGCGTGGTCAGCAAGATAA	For qRT-PCR
flgG-RT-R	GTTGGAGGCTTCTAGCATCG	For qRT-PCR
flgH-RT-F	CCCAGAGCACATTACCGATT	For qRT-PCR
flgH-RT-R	CAGCAAAGGTGTTGGTGTTG	For qRT-PCR
flgA-RT-F	TCGTGGGTTATGGTTTGGTT	For qRT-PCR
flgA-RT-R	TTGACCCGGCTTAGAAAATG	For qRT-PCR
flgJ-RT-F	GATAGCCTGCGTCAGAAAGC	For qRT-PCR
flgJ-RT-R	TCTGCTCATCAAGCATCTGG	For qRT-PCR
flgK-RT-F	AAAGCGATTAAGGCTGACGA	For qRT-PCR
flgK-RT-R	AGATCCAAACCTTGCGATTG	For qRT-PCR
flgL-RT-F	CGATTGTGTTGGTGCGTAAC	For qRT-PCR
flgL-RT-R	CGTAGTTGCCCGATTCATCT	For qRT-PCR
VP0788-RT-F	GCACAGTCTCGTGGTCTTGA	For qRT-PCR
VP0788-RT-R	ACCAACGCCATTGATTCTTC	For qRT-PCR
VP0790-RT-F	AACCGTATCGCAGAAACCAC	For qRT-PCR
VP0790-RT-R	CTTTACCGCTTTCAGCTTGG	For qRT-PCR
VP0791-RT-F	TATTCTGCAACGGCTACGCT	For qRT-PCR
VP0791-RT-R	TTATCCGCGAATGTCGTCGT	For qRT-PCR
VP2229-RT-F	CTTGATGAGCTGCACGGTAA	For qRT-PCR
VP2229-RT-R	CAGGTTCTGGTGCTTTAGGC	For qRT-PCR
VP2232-RT-F	TGACCAGCATGGCAAGTTTA	For qRT-PCR
VP2232-RT-R	AGCTCGCACCTTTAGAACCA	For qRT-PCR
flhG-RT-F	CTAGGTCAGCGTCGAGAACC	For qRT-PCR
flhG-RT-R	TCCGTCGCTTAACACAACCA	For qRT-PCR
flhF-RT-F	GCGAACATGCTGAAGCAATA	For qRT-PCR
flhF-RT-R	GCGCGTAGGATCTAACTTCG	For qRT-PCR
flhA-RT-F	TGGCAACGCATCTAAGTCAG	For qRT-PCR
flhA-RT-R	AGCCAAGGTTTGTACGATGG	For qRT-PCR
fliK-RT-F	GTCGAGAAGAATGGCGAGAG	For qRT-PCR
fliK-RT-R	CCAACTGAGCCTCTGACTCC	For qRT-PCR
fliI-RT-F	TTAAACCCGCTTGACCACGA	For qRT-PCR
fliI-RT-R	CAAAGAGCGCGAAGACCAAG	For qRT-PCR
fliH-RT-F	CCATACCGACAGGCAAACCT	For qRT-PCR
fliH-RT-R	TGTCTGGTCACATGGAAGCC	For qRT-PCR
fliG-RT-F	GCGCCTTGCTTGATCAGTTC	For qRT-PCR
fliG-RT-R	TTGGGGTTTGCCCGACTATG	For qRT-PCR
fliF-RT-F	CGAACCAGTACCCAGCAGAA	For qRT-PCR
fliF-RT-R	GGTGCACCGTACGTTTTTGG	For qRT-PCR
fliE-RT-F	CACTGTGCCCGTTTGCTTAC	For qRT-PCR

fliE-RT-R	TCCGGCGGATGCTTCTATTC	For qRT-PCR
fliS-RT-F	CTCCGCACAAAGTCATTCAA	For qRT-PCR
fliS-RT-R	GGATCATTCTGGTGGTTTGC	For qRT-PCR
VP2256-RT-F	CACTACAAGCTCCGCAACAA	For qRT-PCR
VP2256-RT-R	CTGACGCAGTTTCTGTCCAA	For qRT-PCR
flaG-RT-F	ATCGAACATCCAGCCTTACG	For qRT-PCR
flaG-RT-R	GCCGCTTGAACAGAGAAGTC	For qRT-PCR
VP2258-RT-F	AACGACGGTATCTCCATTGC	For qRT-PCR
VP2258-RT-R	AGGTTGTTTCAGCGATACGG	For qRT-PCR
VP2259-RT-F	AACCGTATCGCAGAAACCAC	For qRT-PCR
VP2259-RT-R	CCTTTACCGCTTTCAGCTTG	For qRT-PCR
VP2261-RT-F	TAACAGCGCAAAAGATGACG	For qRT-PCR
VP2261-RT-R	GAAGCGACAAGTCACGCATA	For qRT-PCR
hcp2-RT-F	TACACGGGTACAGCAATGGA	For qRT-PCR
hcp2-RT-R	TTTTCGCCCTGCTTAGTGAT	For qRT-PCR
VPA1041-RT-F	CAAGGTGATGACGGGGTATC	For qRT-PCR
VPA1041-RT-R	TCGCAAGCTCTAAGGGATGT	For qRT-PCR
VPA1042-RT-F	ATCAACCCTGACTCGAATGG	For qRT-PCR
VPA1042-RT-R	TTTCTTTTTGGCCTGGTGTC	For qRT-PCR
VPA1043-RT-F	TCGAACAGCACGTAGAATCG	For qRT-PCR
VPA1043-RT-R	GTGGCACTTCAGTTTCGTGA	For qRT-PCR
VPA1044-RT-F	TCCTCAACCAAATCCTCGAC	For qRT-PCR
VPA1044-RT-R	GCGTAGTTAGGCGTGTAGCC	For qRT-PCR
VPA1045-RT-F	CCGATGCTCAATGGCTTAAT	For qRT-PCR
VPA1045-RT-R	GCTGCTCTTTACCCAACTGC	For qRT-PCR
VPA1046-RT-F	TCATTGCTGTCTTTTCAACG	For qRT-PCR
VPA1046-RT-R	CTTCGCATTCTGGTGCAGTA	For qRT-PCR
flgN-RT-F	AACTCATGGAACGCAGCAGA	For qRT-PCR
flgN-RT-R	ACCGTGTAATGACTCCAGCG	For qRT-PCR
flgM-RT-F	TCTGCTTGGGCACGATCAAT	For qRT-PCR
flgM-RT-R	TGAAAATCGACAAAGTCGCGG	For qRT-PCR
flgA-RT-F	TACCGACTGGCAAAGGTTGG	For qRT-PCR
flgA-RT-R	AAGTAGACCGCGTAGCCAAG	For qRT-PCR
flgB2-RT-F	GTCCTCGCCAGTAACCTAGC	For qRT-PCR
flgB2-RT-R	TGGTACGGCACCGAATACTG	For qRT-PCR
flgC2-RT-F	CACTCGCTAACGGTGAAGGT	For qRT-PCR
flgC2-RT-R	CTGCCCTAAACGAAGCAAGC	For qRT-PCR
flgD2-RT-F	TCTTGCACAGTACACGGCAT	For qRT-PCR
flgD2-RT-R	TGCGCCACCATCAAAGAGAT	For qRT-PCR
lafK-RT-F	CGCAACAAGTAATCGGTCGC	For qRT-PCR
lafK-RT-R	CCGGATATGTGCGTACGTGA	For qRT-PCR
motY-RT-F	ACCGGCAATCGCCTTCATTA	For qRT-PCR
motY-RT-R	ATGGCAAGGTGCGACACTAA	For qRT-PCR
fliM-RT-F	GACATCCACCCATAAGCACCA	For qRT-PCR

fliM-RT-R	GTAGGCGAAGATCACTGGCA	For qRT-PCR
fliN-RT-F	TGATTTGATGAAAGCGGGCG	For qRT-PCR
fliN-RT-R	CGTCAATCAAGCGAAGACCG	For qRT-PCR
lafA-RT-F	GCAATGCGTAACGCTCAAGA	For qRT-PCR
lafA-RT-R	TGCGCGGTCTTTATCACTGT	For qRT-PCR
lafB-RT-F	AAGTTGAGAGTGCCATGCGT	For qRT-PCR
lafB-RT-R	ACCCGATAGAGCCTTAGCGT	For qRT-PCR
lafC-RT-F	TGGACTCCGGTTACGACTCA	For qRT-PCR
lafC-RT-R	AAACGCTTCGCAGCAAGATG	For qRT-PCR
lafD-RT-F	CGCGCTAAAACGCCATGATT	For qRT-PCR
lafD-RT-R	GCTCACCAGTGGCTTTCTCT	For qRT-PCR
VP0118-RT-F	AGTTCTTTATCCCCCGCAGC	For qRT-PCR
VP0118-RT-R	GGCGAGTGGCAGTGAAGTAT	For qRT-PCR
VP0119-RT-F	CGTCACGTCACTGTCGGTAA	For qRT-PCR
VP0119-RT-R	GCTGAGCTGTTGTTTTCGCA	For qRT-PCR
VP0121-RT-F	GTCTTTGTCCATCGCTTCGC	For qRT-PCR
VP0121-RT-R	AGTGGTACCAAGCCCGAAAG	For qRT-PCR
VP1510-RT-F	TTCCAACCTTGATGGGGACG	For qRT-PCR
VP1510-RT-R	GTCGCTGCCTTCACTCGATA	For qRT-PCR
VP1511-RT-F	CGAACGAAGTGGAGCGAGAA	For qRT-PCR
VP1511-RT-R	TCGCGACGAATTTCCGCTAA	For qRT-PCR
VP1512-RT-F	CCGTAGGGATCTCCTAAAAGGC	For qRT-PCR
VP1512-RT-R	GCGAATGTGTTGAGTTTCACGA	For qRT-PCR
VP1513-RT-F	CCACCAAGCGCGTATTTGTC	For qRT-PCR
VP1513-RT-R	GTGTGCTTCAGCTGGGTTTG	For qRT-PCR
VP1514-RT-F	GCCTTGTATGGCGGTTTGTC	For qRT-PCR
VP1514-RT-R	CGAACGCTTCTTGTTTCGGG	For qRT-PCR
VP1515-RT-F	AATGATGACGGCATTGCTGC	For qRT-PCR
VP1515-RT-R	GTGGTATAGCCCTCTTGCCC	For qRT-PCR
opaR-RT-F	CGCTCGTGAAAACATCGCAA	For qRT-PCR
opaR-RT-R	GGTTAGTGCGGTTGGTAGACA	For qRT-PCR
luxZ-RT-F	TCAGAGGGCGGCATTTTCAT	For qRT-PCR
luxZ-RT-R	AACCGTCAGCGCAACAAAAA	For qRT-PCR
gyrB-RT-F	GAAGCGACAAGTCACGCATA	For qRT-PCR
gyrB-RT-R	CTTCGATGAAGTGACGACGA	For qRT-PCR

Table S3. The differentially expressed genes in Δ*rpoN* compared to wild type

Gene name	Annotation	log2FoldChange (Δ*rpoN*/WT)	P-*value*
VP0006	amino acid ABC transporter ATP-binding protein	1.34	1.39E-15
VP0007	amino acid ABC transporter permease	1.64	1.18E-15
VP0008	amino acid ABC transporter substrate-binding protein	2.36	2.10E-76
VP0024	NADH dehydrogenase subunit II-like protein	-1.18	1.29E-13
VP0051	hypothetical protein	-1.35	3.88E-17
VP0060	multidrug transmembrane resistance signal peptide protein	1.68	1.71E-16
VP0061	multidrug transmembrane resistance signal peptide protein	1.53	5.53E-22
VP0062	phosphogluconate dehydratase	-1.21	0.030745408
VP0067	LysR family transcriptional regulator	-1.26	1.12E-05
VP0071	sensory box/GGDEF family protein	1.17	4.42E-12
VP0074	hypothetical protein	1.35	5.36E-07
VP0085	hypothetical protein	-1.20	0.026811533
VP0111	hypothetical protein	-1.19	0.002476672
VP0117	GGDEF family protein	2.46	5.10E-35
VP0118	nitrogen regulation protein	-1.21	1.83E-08
VP0121	glutamine synthetase	-2.27	6.15E-86
VP0131	hypothetical protein	1.05	8.08E-05
VP0149	hypothetical protein	-1.22	1.46E-14
VP0201	acetyltransferase	1.20	5.70E-07
VP0287	ElaA protein	1.26	0.000166921
VP0291	uroporphyrin-III C-methyltransferase	1.38	1.54E-05
VP0326	hypothetical protein	-1.19	0.016439073
VP0363	glycerol dehydrogenase	-1.11	5.27E-10
VP0365	dihydroxyacetone kinase subunit DhaL	-1.37	3.45E-23
VP0366	phosphoenolpyruvate-protein phosphotransferase	-1.42	2.32E-21
VP0393	hypothetical protein	-2.31	1.81E-45
VP0394	haemagglutinin associated protein	-2.21	1.35E-49
VP0400	transmembrane protein	-1.18	3.54E-14
VP0502	hypothetical protein	-1.28	8.84E-19
VP0556	sigma-54 modulation protein	-1.20	4.26E-09
VP0585	acetoin utilization protein AcuB	-1.40	7.69E-20
VP0625	hypothetical protein	-1.52	5.18E-10
VP0629	homocysteine synthase	-1.04	1.64E-16
VP0652	hypothetical protein	2.86	2.93E-32
VP0672	hypothetical protein	1.02	1.45E-10
VP0675	DNA-binding transcriptional regulator Crl	-1.65	2.47E-32
VP0698	hypothetical protein	-1.07	4.55E-14
VP0699	GGDEF family protein	1.31	1.76E-22
VP0710	PTS system trehalose(maltose)-specific transporter subunits IIBC	-1.68	0.002609259
VP0711	trehalose-6-phosphate hydrolase	-1.26	0.025630694
VP0752	hypothetical protein	-1.06	3.19E-10

VP0766	hypothetical protein	1.51	0.009923442
VP0768	hypothetical protein	-1.96	1.62E-14
VP0769	hypothetical protein	-2.47	8.48E-16
VP0775	flagellar basal-body rod protein FlgB	-2.19	5.27E-47
VP0776	flagellar basal body rod protein FlgC	-2.71	5.43E-36
VP0777	flagellar basal body rod modification protein	-2.88	9.16E-69
VP0778	flagellar hook protein FlgE	-3.41	8.05E-105
VP0780	flagellar basal body rod protein FlgF	-3.96	2.44E-35
VP0781	flagellar basal body rod protein FlgG	-4.47	2.47E-44
VP0782	flagellar basal body L-ring protein	-3.55	1.26E-34
VP0783	flagellar basal body P-ring biosynthesis protein FlgA	-3.40	3.51E-34
VP0784	flagellar rod assembly protein/muramidase FlgJ	-4.09	1.34E-44
VP0785	flagellar hook-associated protein FlgK	-4.95	9.14E-38
VP0786	flagellar hook-associated protein FlgL	-2.83	6.35E-24
VP0788	flagellin	-3.86	1.11E-125
VP0790	flagellin	-2.26	2.03E-07
VP0810	PTS system mannose-specific%2C factor IIC	-1.38	0.001487326
VP0901	hypothetical protein	2.59	4.42E-10
VP0910	C4-dicarboxylate-binding periplasmic protein	-6.52	8.17E-143
VP0911	C4-dicarboxylate transport protein DctQ	-4.38	6.00E-27
VP0912	C4-dicarboxylate transport protein	-4.14	4.59E-75
VP0935	hypothetical protein	1.43	0.000759099
VP0946	cysteine synthase/cystathionine beta-synthase family protein	1.23	9.62E-08
VP0948	hypothetical protein	1.12	1.59E-09
VP0953	hypothetical protein	-1.21	1.79E-13
VP0960	uridine phosphorylase	-1.39	1.48E-28
VP0976	hypothetical protein	1.16	2.80E-05
VP0996	54 kDa polar flagellar sheath protein A	-4.15	7.98E-78
VP1083	ATP-dependent DNA helicase	-1.02	8.40E-11
VP1084	hypothetical protein	-1.27	1.27E-07
VP1088	chemotaxis transducer	-1.45	4.21E-22
VP1111	hypothetical protein	-1.15	0.000780125
VP1119	transcriptional regulator	-1.26	0.004634255
VP1120	short chain dehydrogenase	-1.22	0.011842195
VP1122	hypothetical protein	-1.11	0.025931714
VP1173	phage shock protein A	-1.01	4.36E-10
VP1174	phage shock protein B	-1.19	0.003962859
VP1189	hydroxylamine reductase	-1.37	3.79E-05
VP1207	bifunctional UDP-sugar hydrolase/5'-nucleotidase periplasmic	-1.87	6.42E-62
VP1235	iron-containing alcohol dehydrogenase	-1.09	1.03E-15
VP1241	hypothetical protein	-2.36	3.00E-46
VP1244	response regulator	-1.19	0.000439784
VP1275	formimidoylglutamase	-1.48	6.06E-11
VP1276	imidazolonepropionase	-1.43	2.45E-12

VP1298	cytidine deaminase	-1.95	1.55E-38
VP1315	multidrug resistance protein	1.38	0.001173214
VP1325	hypothetical protein	-1.42	2.79E-05
VP1326	hypothetical protein	-1.24	1.41E-11
VP1376	chemotaxis protein CheY	-1.27	1.12E-10
VP1378	HD-GYP domain-containing protein	1.02	1.20E-17
VP1380	hypothetical protein	1.29	1.71E-12
VP1385	hypothetical protein	-1.13	0.010531085
VP1393	BfdA protein	-1.84	3.17E-05
VP1409	hypothetical protein	1.71	1.13E-17
VP1428	hypothetical protein	-1.12	0.015738044
VP1447	anaerobic dimethyl sulfoxide reductase subunit A	1.01	9.04E-12
VP1474	capsule transport protein OtnA	-1.83	0.000143036
VP1499	ABC transporter permease	-1.83	2.76E-12
VP1501	hypothetical protein	-1.45	7.63E-14
VP1503	sensor histidine kinase	-1.25	1.71E-08
VP1508	hypothetical protein	-1.84	1.03E-08
VP1509	hypothetical protein	-2.04	2.31E-25
VP1510	(Fe-S)-binding protein	-4.02	1.29E-22
VP1511	formate dehydrogenase-specific chaperone	-4.75	1.92E-71
VP1512	hypothetical protein	-3.17	7.58E-10
VP1513	formate dehydrogenase large subunit	-4.60	3.99E-151
VP1514	formate dehydrogenase%2C iron-sulfur subunit	-3.93	2.50E-22
VP1515	formate dehydrogenase%2C cytochrome b556 subunit	-4.05	9.61E-74
VP1516	hypothetical protein	-4.04	1.35E-39
VP1517	Rhs-family protein	-1.66	1.00E-31
VP1518	hypothetical protein	-1.02	3.88E-17
VP1601	dihydroorotate dehydrogenase 2	1.01	2.94E-12
VP1633	RTX toxin	-1.60	1.86E-36
VP1634	agglutination protein	-1.04	8.55E-08
VP1635	outer membrane protein	-1.45	1.39E-12
VP1659	hypothetical protein	1.21	5.04E-15
VP1660	type III secretion regulator	1.29	0.001791353
VP1664	type III secretion protein	1.34	0.010663116
VP1665	type III secretion protein	1.42	8.75E-05
VP1666	hypothetical protein	1.31	0.000374227
VP1667	outer membrane protein PopN	1.49	2.81E-17
VP1668	type III secretion system ATPase	1.33	1.42E-12
VP1669	type III secretion protein YscO	1.59	0.000150664
VP1670	translocation protein in type III secretion	1.42	2.75E-06
VP1675	translocation protein in type III secretion	1.19	0.0219073
VP1680	hypothetical protein	1.13	4.31E-10
VP1682	hypothetical protein	1.47	4.39E-08
VP1686	adenosine monophosphate-protein transferase VopS	1.25	2.65E-13

VP1687	type III chaperone	1.21	8.61E-08
VP1689	type III secretion protein	1.38	0.002388787
VP1692	type III export protein	1.88	5.48E-07
VP1694	type III export protein YscF	1.25	0.000164246
VP1697	type III export apparatus protein NosA	1.08	0.015535225
VP1698	hypothetical protein	1.49	3.79E-30
VP1701	exoenzyme S synthesis protein C	1.96	4.96E-15
VP1702	hypothetical protein	1.96	3.30E-10
VP1703	aldehyde dehydrogenase	-4.86	4.71E-65
VP1711	response regulator	-1.32	3.80E-09
VP1749	hypothetical protein	-1.57	2.30E-22
VP1779	glutamine amidotransferase	1.25	0.007430945
VP1781	glutamine synthetase	1.67	6.69E-05
VP1782	hypothetical protein	1.56	8.57E-05
VP1784	hypothetical protein	-1.49	0.000426166
VP1792	hypothetical protein	-1.14	0.006974001
VP1823	hypothetical protein	-1.05	0.000274812
VP1827	spermine/spermidine acetyltransferase BltD	-1.07	0.012336579
VP1851	hypothetical protein	-1.23	5.33E-19
VP1861	hypothetical protein	-1.12	5.29E-09
VP1881	hypothetical protein	2.48	4.31E-22
VP1892	methyl-accepting chemotaxis protein	-1.18	4.87E-11
VP1897	hypothetical protein	-1.98	2.27E-28
VP1898	hypothetical protein	-1.07	9.50E-13
VP1907	LuxR family transcriptional regulator	-1.45	4.36E-12
VP1915	hypothetical protein	1.40	3.15E-16
VP1918	hypothetical protein	1.21	0.000759305
VP1919	hypothetical protein	1.15	0.000109182
VP1920	iron-regulated protein A	1.92	3.14E-25
VP1925	NrfE protein	-1.09	0.006531
VP1926	formate dependent nitrate reductase NrfD	-1.31	2.75E-14
VP1966	proton/glutamate symporter	1.54	4.11E-31
VP1967	acyl-homoserine-lactone synthase OpaM	-1.31	1.34E-15
VP1968	sensor protein LuxN	-1.66	1.19E-37
VP1969	hypothetical protein	-1.40	3.19E-08
VP1977	hypothetical protein	-1.20	8.53E-08
VP1979	hypothetical protein	-1.21	1.21E-06
VP1987	hypothetical protein	-1.97	2.31E-35
VP2011	tetrathionate reductase subunit B	1.06	1.09E-18
VP2015	cytochrome c	1.78	5.47E-29
VP2016	hypothetical protein	1.53	3.47E-18
VP2041	hypothetical protein	-1.05	6.16E-10
VP2110	hypothetical protein	1.04	0.004503097
VP2119	hypothetical protein	-1.72	0.001183582

VP2120	short chain dehydrogenase	-1.27	2.22E-06
VP2121	bifunctional acetaldehyde-CoA/alcohol dehydrogenase	-1.08	1.07E-10
VP2159	methyl-accepting chemotaxis transmembrane protein	1.25	3.92E-05
VP2161	hypothetical protein	1.63	1.53E-05
VP2162	hypothetical protein	-1.56	0.000476209
VP2165	hypothetical protein	1.06	0.000255831
VP2173	hypothetical protein	-1.29	6.87E-06
VP2174	regulatory protein	-1.01	0.001048408
VP2197	hypothetical protein	1.05	0.001049892
VP2200	hypothetical protein	1.31	0.001657683
VP2210	RNA polymerase sigma factor	1.08	3.39E-06
VP2229	chemotaxis protein CheA	-1.11	1.54E-15
VP2232	flagellar biosynthesis sigma factor	-1.83	6.02E-40
VP2233	flagellar biosynthesis protein FlhG	-3.71	3.51E-115
VP2234	flagellar biosynthesis regulator FlhF	-5.35	6.38E-154
VP2235	flagellar biosynthesis protein FlhA	-3.60	3.78E-78
VP2244	polar flagellar hook-length control protein FliK	-1.86	4.68E-24
VP2248	flagellar motor switch protein G	-1.40	4.17E-14
VP2251	FlaM	-1.23	1.20E-18
VP2254	flagellar protein FliS	-3.08	2.88E-16
VP2256	flagellar capping protein	-3.66	5.73E-44
VP2257	flagellar protein FlaG	-3.33	4.50E-28
VP2258	flagellin	-3.00	2.95E-69
VP2259	flagellin	-3.33	7.63E-23
VP2261	flagellin	-2.17	8.90E-07
VP2282	hypothetical protein	1.00	9.05E-11
VP2290	sulfate permease	-1.04	1.05E-10
VP2327	hypothetical protein	1.47	0.000647099
VP2372	hypothetical protein	-1.47	4.09E-20
VP2388	glycerol-3-phosphate dehydrogenase	-1.44	0.013120153
VP2394	sodium:galactoside symporter family protein	1.87	2.05E-33
VP2491	iron(III) ABC transporter periplasmic iron-compound-binding protein	1.69	1.26E-40
VP2492	ammonium transporter	-1.47	5.85E-05
VP2516	OpaR protein	-1.64	1.41E-31
VP2631	HD-GYP domain-containing protein	1.71	4.01E-17
VP2636	PTS system cellobiose-specific transporter subunit Iic transporter subunit	-1.31	0.001933048
VP2637	PTS system cellobiose-specific transporter subunit IIB	-1.88	0.000168365
VP2638	deacetylase DA1	-1.58	2.72E-06
VP2670	RNA polymerase factor sigma-54	-8.41	5.15E-113
VP2671	sigma-54 modulation protein	-1.41	8.07E-18
VP2699	MSHA biogenesis protein MshF	-1.55	5.03E-16
VP2722	sulfite reductase (NADPH) flavoprotein subunit alpha	1.18	1.78E-10

VP2725	phage shock protein G	-2.10	4.70E-06
VP2769	bacterioferritin-associated ferredoxin	1.14	9.58E-05
VP2799	extracellular nuclease-like protein	-1.06	8.40E-11
VP2811	sodium-type polar flagellar protein MotX	-1.24	0.000650442
VP2843	fumarate reductase subunit D	-1.22	0.005377588
VP2844	hypothetical protein	-2.00	9.82E-06
VP2864	anaerobic C4-dicarboxylate transporter	-1.07	3.90E-11
VP2876	hypothetical protein	1.03	3.06E-08
VP2888	sensory box/GGDEF family protein	1.54	2.43E-06
VP2891	lysine/cadaverine antiporter	1.13	0.035944955
VP2900	hypothetical protein	-1.64	2.84E-29
VP2933	hypothetical protein	1.27	0.003212014
VP2964	hypothetical protein	-1.08	1.58E-12
VP2979	GGDEF family protein	-1.57	7.56E-21
VP3009	AraC family transcriptional regulator	1.30	1.37E-13
VP3010	hypothetical protein	1.20	4.80E-17
VP3012	hypothetical protein	-1.71	1.16E-06
VP3019	multidrug resistance protein	1.20	5.48E-13
VP3050	hypothetical protein	-1.14	1.23E-09
VPA0035	sodium/glutamate symporter	1.02	3.79E-07
VPA0056	hypothetical protein	1.24	2.14E-05
VPA0058	hypothetical protein	-1.01	3.58E-06
VPA0104	ring-cleaving dioxygenase	-1.87	1.32E-05
VPA0109	hypothetical protein	1.14	0.012661022
VPA0115	hypothetical protein	1.02	0.000100009
VPA0122	hypothetical protein	-1.15	0.019076086
VPA0148	transcriptional regulator CpxR	1.14	0.005724673
VPA0149	two-component system sensor kinase	1.18	0.0004456
VPA0151	hypothetical protein	1.31	1.05E-05
VPA0156	hypothetical protein	1.17	5.34E-08
VPA0166	outer membrane protein	-1.45	3.91E-29
VPA0188	hypothetical protein	-2.81	1.81E-08
VPA0189	hypothetical protein	-2.44	2.52E-27
VPA0197	hypothetical protein	1.16	0.000749851
VPA0213	hypothetical protein	1.16	0.007511307
VPA0251	LysR family transcriptional regulator	2.69	6.71E-47
VPA0252	hypothetical protein	3.46	2.02E-33
VPA0296	oxidoreductase protein	-2.77	2.16E-62
VPA0300	signal peptide protein	2.22	1.60E-08
VPA0328	elongation factor G	-1.13	3.25E-19
VPA0345	hypothetical protein	-1.44	0.003459281
VPA0347	hypothetical protein	-2.09	2.82E-26
VPA0348	hypothetical protein	-1.43	2.86E-13
VPA0358	LuxR family transcriptional regulator	-1.25	3.28E-06

VPA0361	hypothetical protein	1.11	0.032912147
VPA0382	sorbitol-6-phosphate 2-dehydrogenase	-1.33	0.003255799
VPA0383	hypothetical protein	-1.35	0.007790699
VPA0384	hypothetical protein	-1.03	0.008579881
VPA0392	hypothetical protein	1.19	1.20E-13
VPA0442	hypothetical protein	-1.06	1.47E-15
VPA0443	hypothetical protein	-1.43	1.04E-17
VPA0444	hypothetical protein	-1.41	9.97E-06
VPA0445	hypothetical protein	-1.18	0.007601007
VPA0446	methylamine utilization protein MauG	-1.23	4.56E-09
VPA0449	serine proteinase	1.40	7.52E-08
VPA0450	hypothetical protein	1.39	1.17E-08
VPA0451	hypothetical protein	1.35	0.010446488
VPA0458	hypothetical protein	-1.08	9.66E-07
VPA0460	PAS factor	-1.49	9.53E-15
VPA0478	oxidoreductase	-1.61	9.44E-25
VPA0482	outer membrane cation efflux protein	1.61	1.49E-05
VPA0486	hypothetical protein	1.09	0.022924965
VPA0491	methyl-accepting chemotaxis protein	1.96	6.77E-26
VPA0500	PTS system mannitol-specific transporter subunit II	1.55	2.33E-05
VPA0510	hypothetical protein	1.62	0.002705592
VPA0511	methyl-accepting chemotaxis protein	1.30	1.37E-06
VPA0518	GGDEF family protein	-1.21	6.56E-10
VPA0548	hypothetical protein	-3.08	1.82E-19
VPA0559	hypothetical protein	-1.34	4.75E-07
VPA0566	alcohol dehydrogenase	-1.02	2.22E-11
VPA0585	tryptophan synthase subunit beta	-1.12	2.82E-17
VPA0612	chemotaxis transducer	1.12	1.82E-11
VPA0627	cytochrome o ubiquinol oxidase subunit II	1.45	0.00036702
VPA0657	iron(III) ABC transporter periplasmic iron-compound-binding protein	1.42	1.13E-10
VPA0658	iron(III) ABC transporter permease	1.25	0.001295329
VPA0659	iron(III) ABC transporter permease	1.30	0.00145376
VPA0660	iron(III) ABC transporter ATP-binding protein	1.15	0.000365534
VPA0711	hypothetical protein	-1.08	4.19E-05
VPA0733	LysR family transcriptional regulator	1.17	0.001521171
VPA0743	response regulator VieB	1.05	0.03022484
VPA0747	MSHA pilin protein MshA	3.23	3.87E-61
VPA0780	hypothetical protein	-1.45	7.55E-28
VPA0811	PTS system fructose-specific transporter subunit IIBC	-1.31	1.78E-05
VPA0812	1-phosphofructokinase	-1.61	0.000783696
VPA0813	bifunctional PTS system fructose-specific transporter subunit IIA/HPr protein	-1.19	0.000866908
VPA0825	phosphoglycerate transport regulatory protein PgtC	1.28	1.20E-12

VPA0853	membrane protein YcdZ	-2.74	9.86E-57
VPA0878	hypothetical protein	-1.22	5.68E-14
VPA0882	heme transport protein HutA	1.03	0.000118665
VPA0914	hypothetical protein	-3.11	3.26E-40
VPA0946	hypothetical protein	-1.66	0.005366517
VPA0969	hypothetical protein	-1.56	0.000134984
VPA0970	hypothetical protein	-1.48	0.000439784
VPA0980	hypothetical protein	1.11	0.040029605
VPA0981	anaerobic C4-dicarboxylate transporter	-1.10	6.78E-15
VPA1018	lipoprotein Blc	-1.00	0.000470775
VPA1020	hypothetical protein	-1.21	0.007260041
VPA1024	hypothetical protein	-1.40	9.00E-05
VPA1026	hypothetical protein	-2.29	3.12E-22
VPA1027	hypothetical protein	-3.85	2.82E-75
VPA1028	ClpA/B-type chaperone	-1.40	6.23E-08
VPA1029	hypothetical protein	-2.10	2.19E-05
VPA1030	hypothetical protein	-1.56	1.11E-05
VPA1032	hypothetical protein	-2.81	1.45E-07
VPA1033	hypothetical protein	-2.08	2.22E-06
VPA1034	hypothetical protein	-3.48	2.32E-29
VPA1035	hypothetical protein	-2.52	2.76E-08
VPA1036	hypothetical protein	-2.43	6.12E-13
VPA1037	phosphoprotein phosphatase	-2.16	1.39E-07
VPA1038	hypothetical protein	-3.21	1.84E-15
VPA1039	hypothetical protein	-2.66	4.48E-40
VPA1040	hypothetical protein	-3.03	3.40E-17
VPA1041	hypothetical protein	-3.21	7.54E-23
VPA1042	hypothetical protein	-3.71	4.16E-21
VPA1043	hypothetical protein	-3.53	7.48E-51
VPA1044	hypothetical protein	-3.43	2.03E-35
VPA1045	hypothetical protein	-1.72	9.84E-15
VPA1046	hypothetical protein	-2.27	1.48E-14
VPA1051	glutathione S-transferase	-1.44	0.018936776
VPA1081	hypothetical protein	1.07	1.36E-08
VPA1097	hypothetical protein	-1.08	0.000746215
VPA1117	3-ketoacyl-ACP reductase	-1.16	1.29E-08
VPA1118	3-hydroxyisobutyrate dehydrogenase	-1.09	1.36E-08
VPA1119	enoyl-CoA hydratase/isomerase	-1.05	1.82E-08
VPA1121	acyl-CoA dehydrogenase	-1.26	2.23E-10
VPA1122	aldehyde dehydrogenase	-1.15	1.06E-09
VPA1123	acyl-CoA thiolase	-1.05	0.021089204
VPA1126	acyl-CoA carboxyltransferase beta chain	-1.07	1.47E-07
VPA1135	acetyltransferase	-1.30	1.31E-05
VPA1147	phenylacetate-CoA ligase	-1.13	8.29E-11

VPA1148	ABC transporter ATP-binding protein	-1.41	2.60E-11
VPA1151	ABC transporter membrane spanning protein	-1.02	1.40E-08
VPA1152	long-chain-fatty-acid-CoA ligase	-1.19	2.71E-15
VPA1153	ABC transporter ATP-binding protein	-1.14	2.46E-12
VPA1186	outer membrane protein OmpA	-2.89	3.62E-116
VPA1203	hypothetical protein	-1.14	9.21E-05
VPA1204	acetyl-CoA acetyltransferase	-2.33	2.35E-14
VPA1205	acetoacetyl-CoA reductase	-2.23	1.57E-09
VPA1210	hypothetical protein	1.17	1.27E-14
VPA1297	hypothetical protein	1.43	0.004661889
VPA1345	hypothetical protein	-1.19	3.57E-10
VPA1352	hypothetical protein	-1.29	0.013203315
VPA1407	hypothetical protein	-1.32	4.44E-09
VPA1409	hypothetical protein	-1.38	3.41E-06
VPA1413	hypothetical protein	-1.15	0.036408997
VPA1423	transcriptional regulator	-1.20	5.66E-05
VPA1434	hemolysin secretion ATP-binding protein	1.24	7.18E-12
VPA1435	iron(III) compound receptor	1.93	5.53E-29
VPA1436	iron(III) ABC transporter ATP-binding protein	2.38	3.35E-08
VPA1437	iron(III) ABC transporter periplasmic iron-compound-binding protein	1.63	0.000983719
VPA1438	iron-hydroxamate transporter permease subunit	1.11	0.000690946
VPA1444	transport protein	-1.21	5.94E-08
VPA1445	secreted calcium-binding protein	-1.94	4.50E-31
VPA1446	LuxR family transcriptional regulator	-1.02	1.65E-06
VPA1449	methyl-accepting chemotaxis protein	-1.53	2.80E-05
VPA1457	GGDEF family protein	-1.19	8.91E-07
VPA1461	phosphate ABC transporter periplasmic phosphate-binding protein	1.04	0.028072106
VPA1462	methyl-accepting chemotaxis protein	1.99	5.82E-16
VPA1475	purine nucleoside phosphorylase	-1.47	2.29E-18
VPA1476	hypothetical protein	-1.70	1.34E-22
VPA1507	CsuA protein	-2.59	1.18E-06
VPA1508	hypothetical protein	-1.04	0.039227635
VPA1509	L-threonine 3-dehydrogenase	-1.16	1.10E-07
VPA1571	acetyltransferase	-1.45	4.42E-12
VPA1572	hypothetical protein	-3.10	4.29E-105
VPA1576	transmembrane protein	1.50	1.05E-05
VPA1602	capsular polysaccharide transport protein	-1.74	4.62E-26
VPA1603	phosphatase	-2.27	5.17E-31
VPA1604	tyrosine kinase	-1.72	2.47E-25
VPA1611	carboxypeptidase G2	-1.67	1.30E-21
VPA1612	hypothetical protein	-1.30	3.37E-11
VPA1619	4-alpha-glucanotransferase	-2.00	7.58E-23

VPA1620	maltodextrin phosphorylase	-1.80	3.45E-23
VPA1634	putrescine transporter	1.69	0.000127588
VPA1635	ornithine decarboxylase	2.27	1.77E-08
VPA1638	pullulanase	-2.78	7.32E-71
VPA1642	CymC protein	-1.46	2.39E-08
VPA1644	maltoporin	-1.52	6.19E-30
VPA1648	hypothetical protein	-1.38	1.44E-24
VPA1652	iron-dicitrate transporter ATP-binding subunit	1.21	0.027655236
VPA1656	ferric vibrioferrin receptor	1.45	4.53E-06
VPA1657	ferric siderophore receptor-like protein	1.23	1.97E-05
VPA1670	collagenase family protease	1.18	0.000632758
VPA1703	small integral C4-dicarboxylate membrane transport protein	1.16	0.020141932
