# Supplementary figures and images for: Characterization of the RpoN regulon reveals the regulation of motility, T6SS2 and metabolism in Vibrio parahaemolyticus
Source: Front Microbiol. 2022 Dec 22;13:1025960. doi: 10.3389/fmicb.2022.1025960 (PMC9817140; doi:10.3389/fmicb.2022.1025960)

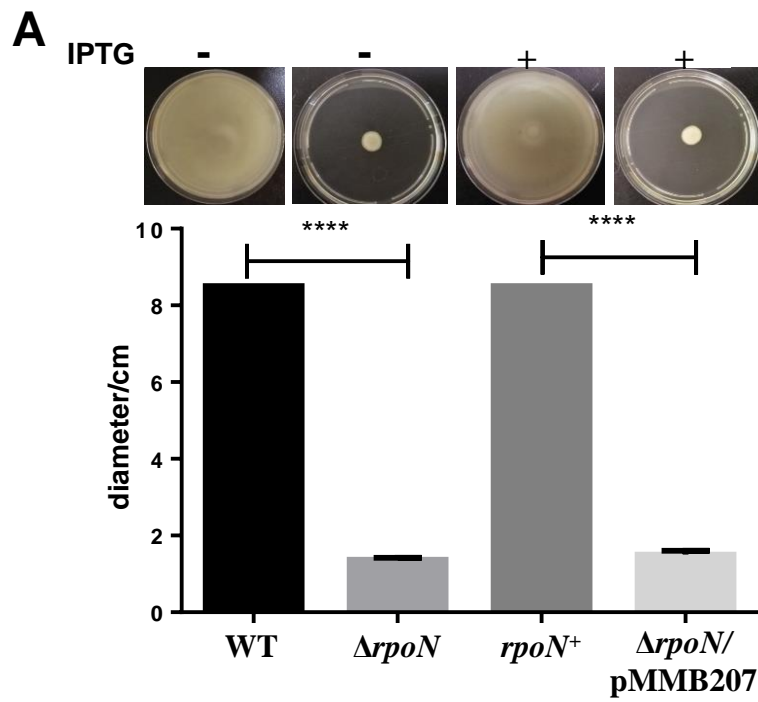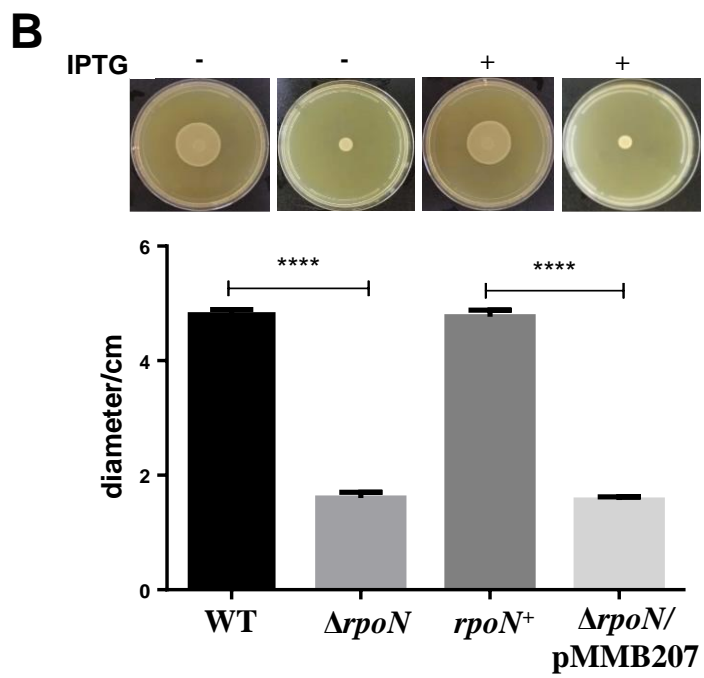

**Figure S1**

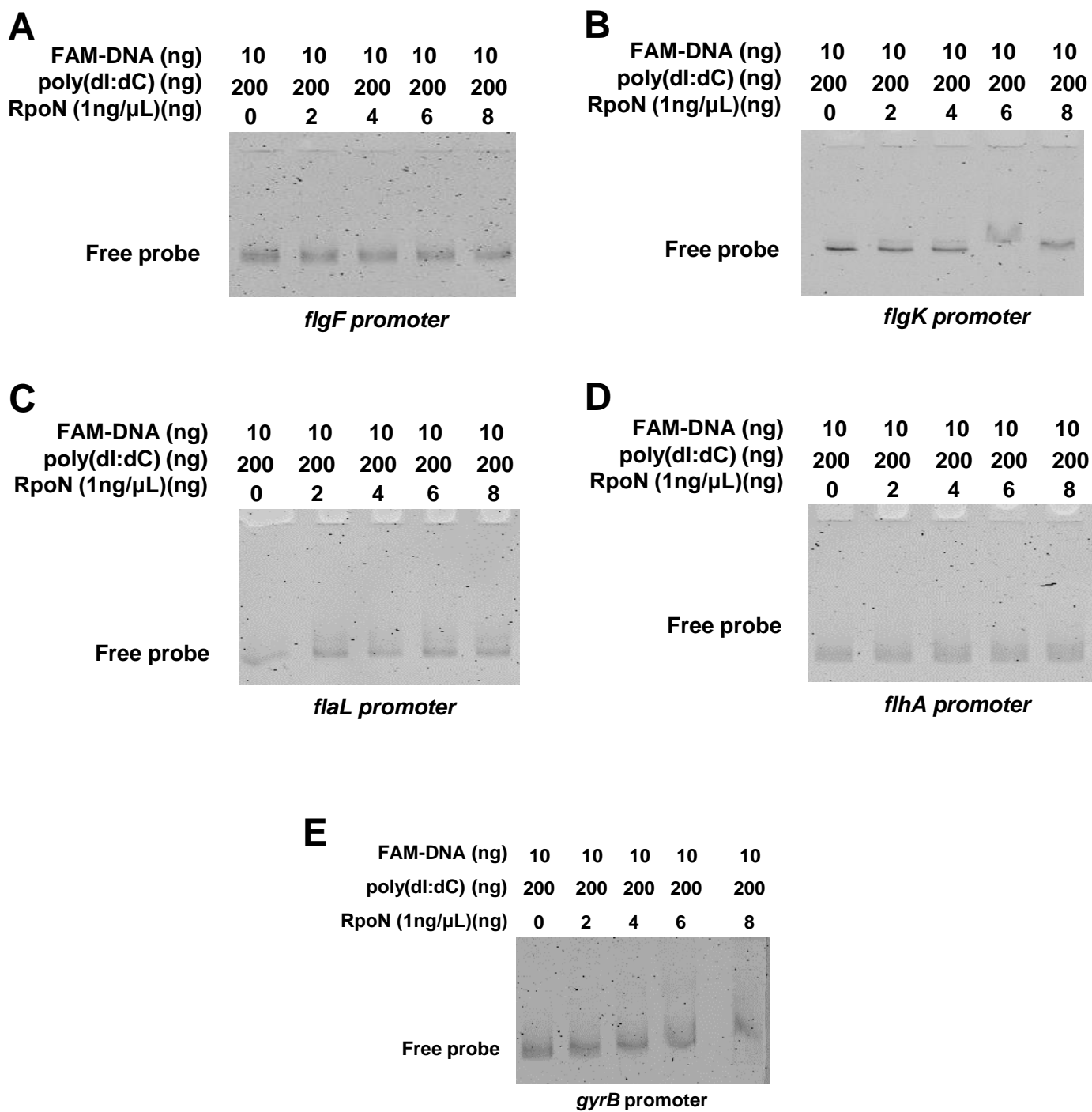

**Figure S2**

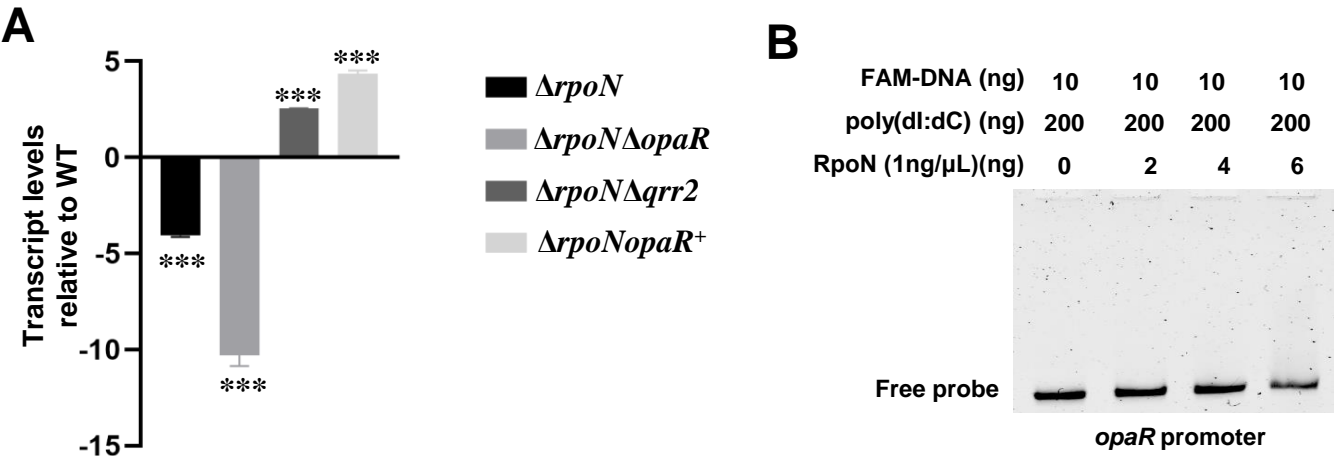

Figure S3

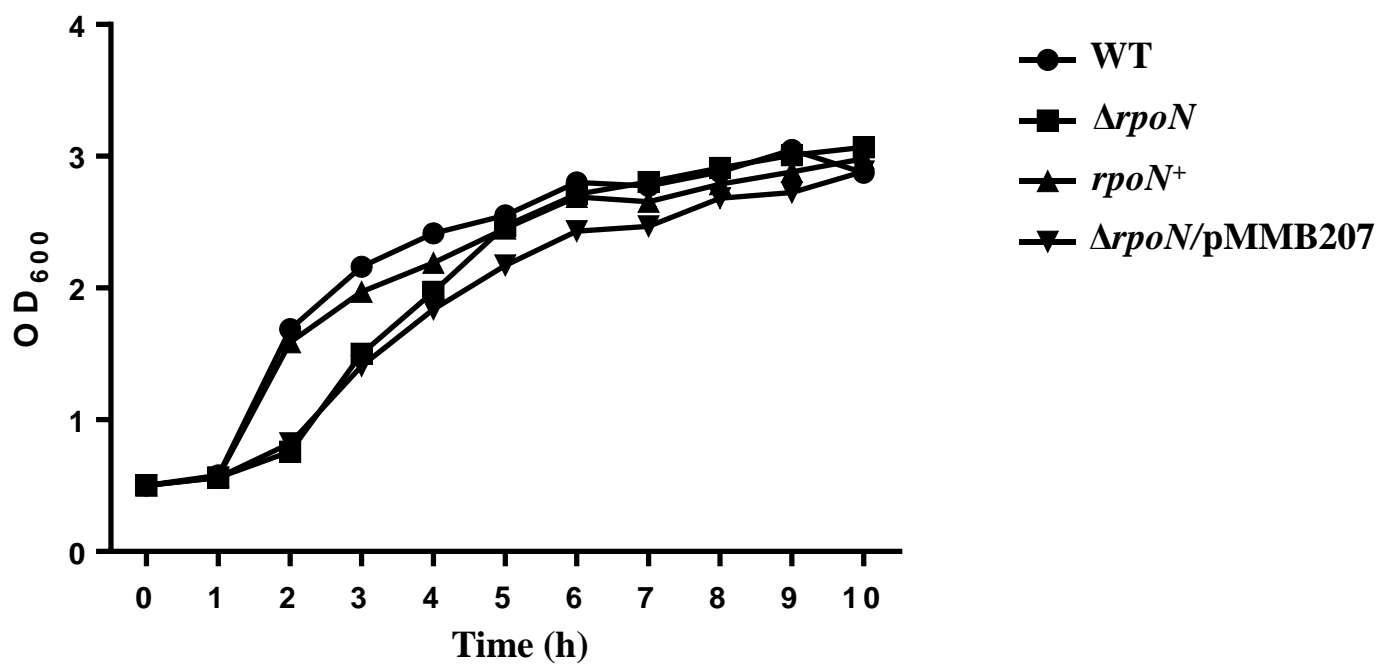

**Figure S4**

Supplement: Supplementary file 2 [file Data_Sheet_2.PDF]
